# High Temperature Stability of Onion-Like Carbon vs Highly Oriented Pyrolytic Graphite

**DOI:** 10.1371/journal.pone.0105788

**Published:** 2014-08-25

**Authors:** Alessandro Latini, Massimo Tomellini, Laura Lazzarini, Giovanni Bertoni, Delia Gazzoli, Luigi Bossa, Daniele Gozzi

**Affiliations:** 1 Dipartimento di Chimica, Università di Roma La Sapienza, Roma, Italy; 2 Dipartimento di Scienze e Tecnologie Chimiche, Università di Roma Tor Vergata, Roma, Italy; 3 IMEM – CNR, Parma, Italy; Jacobs University Bremen, Germany

## Abstract

The thermodynamic stability of onion-like carbon (OLC) nanostructures with respect to highly oriented pyrolytic graphite (HOPG) was determined in the interval 765–1030 K by the electromotive force (*emf*) measurements of solid electrolyte galvanic cell: (Low) Pt|Cr_3_C_2_,CrF_2_,OLC|CaF_2_s.c.|Cr_3_C_2_,CrF_2_,HOPG|Pt (High). The free energy change of transformation HOPG = OLC was found positive below 920.6 K crossing the zero value at this temperature. Its trend with temperature was well described by a 3^rd^ degree polynomial. The unexpected too high values of 
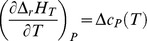
 jointly to the HR-TEM, STEM and EELS evidences that showed OLC completely embedded in rigid cages made of a Cr_3_C_2_/CrF_2_ matrix, suggested that carbon in the electrodes experienced different internal pressures. This was confirmed by the evaluation under constant volume of 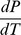 by the 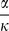 ratio for OLC (0.5 MPa K^−1^) and HOPG (8 Pa K^−1^) where 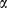 and 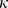 are the isobaric thermal expansion and isothermal compressibility coefficients, respectively. The temperature dependency of the pressure was derived and utilized to calculate the enthalpy and entropy changes as function of temperature and pressure. The highest value of the internal pressure experienced by OLC was calculated to be about 7 GPa at the highest temperature. At 920.6 K, 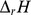 and 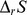 values are 95.8 kJ mol^−1^ and 104.1 JK^−1^ mol^−1^, respectively. The surface contributions to the energetic of the system were evaluated and they were found negligible compared with the bulk terms. As a consequence of the high internal pressure, the values of the enthalpy and entropy changes were mainly attributed to the formation of carbon defects in OLC considered as multishell fullerenes. The change of the carbon defect fraction is reported as a function of temperature.

## Introduction

The modifications of the internal arrangements and related energies of single wall carbon nanotubes (SWCNTs) in bundles were studied [1] by our group in the particular situation where their dilatation due to the high temperatures was hindered being the bundles embedded in a matrix much less dilatable. This generates high internal pressures, which are a direct consequence of the increase of the internal energy of the system producing changes in the SWCNT configuration inside the bundle and deformation of SWCNTs too.

The knowledge of the thermodynamic stability of nanostructures is a fundamental aspect to design new nanostructured materials as well as to forecast their behaviour. Most of the information on this subject comes from computational studies [2] [3] [4] [5] [6] and to a lesser extent by experimental works [7] [8] [9]. To the best of author knowledge and with the exception of reference [10] [11] [12] there are no articles dealing with thermodynamic measurements at high temperature on carbon nanostructures.

On the other hand, the 3D carbon phase diagram, where the third axis is the particle size, is still under consideration. The contribution of the surface energy to the bulk Gibbs free energy per atom of a cluster of *n* atoms in a given phase is strongly affected by the number of atoms itself. In general, a review of the literature regarding the structure of carbon nanoparticles [13] highlights that at sizes below 1.8 nm, other carbon forms are abundant, such as fullerenes and onion-like carbon (OLC). Therefore, Kuznetsov et al. [14] suggested to assign a corresponding region of the phase diagram to closed-shell *sp* ^2^-bonded nanocarbons. In their phase diagram the regions of stability are specifically indicated for fullerenes and OLC for *n* = 10 to 10^2^ atoms. The complete and thorough vision of the phase diagrams of these nanostructures in comparison with the phase diagram of the corresponding bulk materials has not been realized yet. There is still a great deal of work to be done in the construction of a complete *P–T–n* phase diagram of carbon. It is likely however that knowledge gained from theoretical and computational studies [15] of nanocarbon stability plays an important role in the conception of such a diagram, with thermodynamic treatments of phase equilibrium showing the right way for pursuing the final goal.

Onion-like carbon are another class of carbon nanostructures, which are not exhaustively investigated yet though some published results envisage interesting applications such as an easy route to produce diamond nanoparticles [7] [8] [16] as well as to use them as reaction cells on the nanoscale [9].

The scope of this work is to study, in conditions of reversibility, the thermodynamics of the high temperature transformation under volume constraint from highly oriented pyrolytic graphite (HOPG) to OLC.

## Experimental

Since the experimental set-up was published elsewhere [10] [11] [12] and there is the will of the authors to give in this paper more room to the chemical thermodynamics of our experiment and discussion of the results, we decided to describe more in details the wide experimental part in the Supporting Information S1. A list of all the aspects that took part to the whole experimentation of this work, which are reported in the Supporting Information S1, follows:

### 1. Starting materials for the electrode preparation

1.1. HOPG, OLC, Cr_3_C_2_, CrF_2_
1.2. Characterization
  1.2.1. Thermogravimetry (TG) and Differential Thermal Analysis (DTA)
  1.2.2. x-ray diffraction (XRD)
  1.2.3. microRaman (mR)
  1.2.4. High Resolution Transmission Electron Spectroscopy (HR-TEM)
  1.2.5. Electron Energy Loss Spectroscopy (EELS)
  1.2.6. X-ray Photoelectron Spectroscopy (XPS)
1.3. Preparation of electrodes and cell assembly
  1.3.1. Electrodes
  1.3.2. Cell assembly
1.4. Experimental apparatus for the *emf* measurements
1.5. Procedure adopted for the *emf* measurements and their data acquisition
1.6. Analysis of electrodes before and after experiment
  1.6.1. X-ray diffraction (XRD)
  1.6.2. microRaman spectroscopy (mR)
  1.6.3. EELS

## Results and Discussion

In order to satisfy the requirement of reversibility, the experimental technique utilized is the electromotive force (*emf*) measurement as function of temperature of galvanic cells, with CaF_2_ single crystal as solid electrolyte. Since long time this technique was utilized in our laboratory and it is well known that the method is one of the best way to achieve reliable thermodynamic data.

The galvanic cell:

(Low) Pt|Cr_3_C_2_,CrF_2_,OLC|CaF_2_s.c.|Cr_3_C_2_,CrF_2_,HOPG|Pt (High) (A)

was assembled to perform the *emf* vs *T* measurement. Cell A is a fluorine concentration cell being the solid electrolyte an ionic conductor by F^-^ Frenkel type defects. The positive electrode is the electrode where the chemical potential of F_2_(g) is higher and the chemical potential of carbon is lower. This implies that 
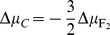
. The cell reaction can be written as:
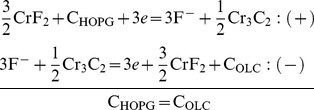
(B)The main advantage of this kind of cells is that no supplementary thermodynamic data are necessary to derive the thermodynamics of the cell reaction. Only the *emf vs T* experimental data are necessary to obtain the reaction Gibbs free energy change, 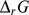. The reaction changes of enthalpy, 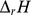, and entropy, 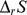, can be also obtained as shown in the followings.

**Figure 1:**
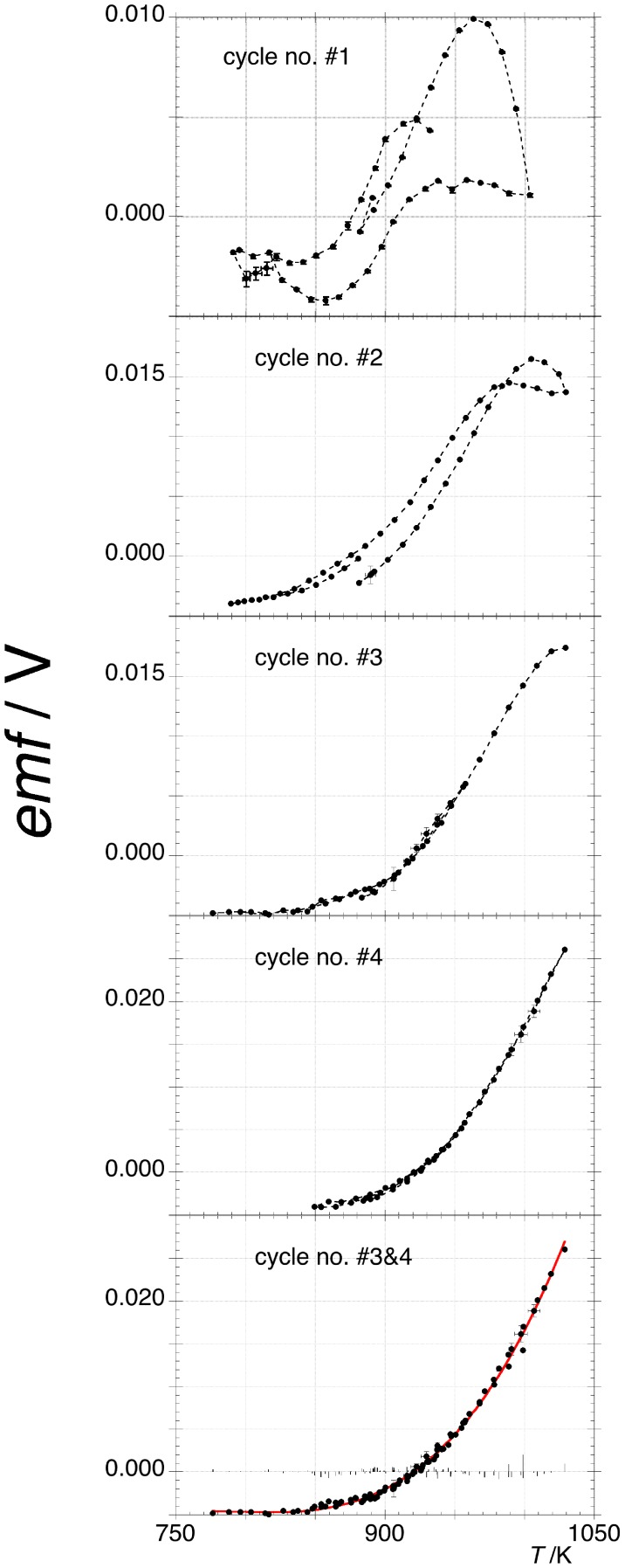
The electromotive force (*emf*) vs temperature of galvanic cell A. The progressive disappearing hysteresis is shown from 1^st^ to 3^rd^ thermal cycle (see §1.4 of the Supporting Information S1). The bottom plot combines the 3^rd^ and 4^th^ cycles where the red curve is the best fitting curve given by a 3^rd^ degree polynomial (see Table 1). The residuals of fit are also reported. Most of the error bars are within the size of the experimental points.

The adopted experimental procedure requires accumulating many isothermal and stationary values of *emf* through several stair shaped thermal cycles each one composed of tens of isotherms. Figure 1 shows the typical behaviour, almost equal for each one, of five tested cells. The first thermal cycles are characterized by hysteresis that tends to disappear. The presence of a vanishing hysteresis in the *emf* trend demonstrates that the whole system of the carbon shells in the OLC and OLC themselves change to reach new stable configurations that convert reciprocally and reversibly as function of temperature. These findings are qualitatively and quantitatively similar to the behaviour we found in the bundles of SWCNTs [1]. The main difference with the present experiment is the final shape of the *emf* vs *T* curve that is characterized here by an exponential growth-like behaviour.

**Figure 2:**
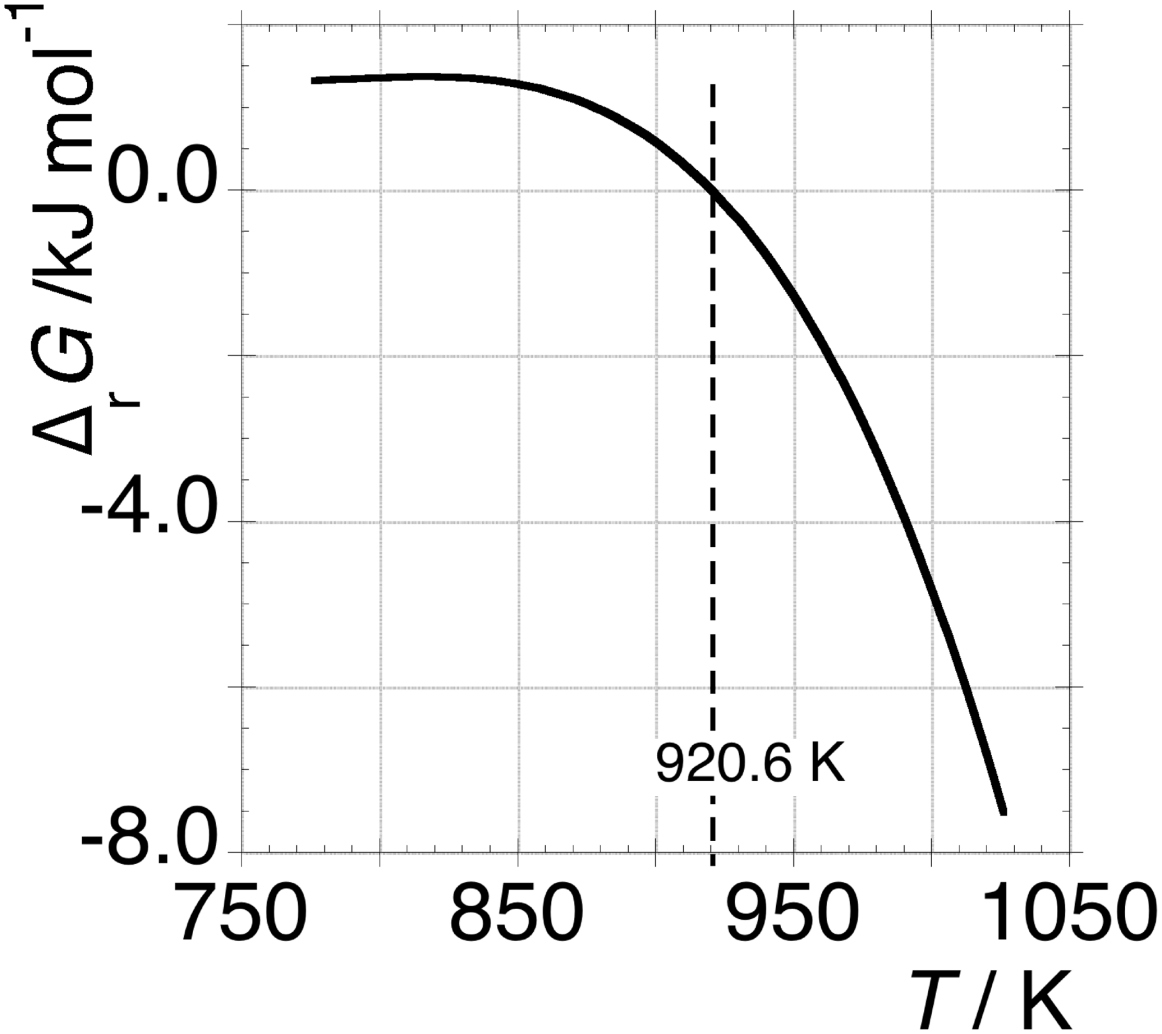
Gibbs free energy change for C_HOPG_ = C_OLC_ transformation calculated according to eq 1. At 920.6 K, the curve crosses zero.

The trend of the Gibbs free energy change is given by:
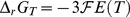
(1)being 3 the number of moles of electrons exchanged in reaction B and 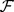 the Faraday constant. The curve 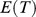 is well fit by the 3^rd^ degree polynomial:



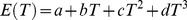
(2).The coefficients and related errors of fit of *emf* vs *T* are given in Table 1. The representation of eq 1 is given in Fig. 2 where at 920.6 K the equality of the chemical potential of carbon 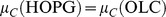
is satisfied.

**Table 1:** Coefficients and related errors of *emf* vs *T* polynomial fit: 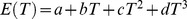.

	*a*/V	*b*/VK^−1^	*c*/VK^−2^	*d*/VK^−3^
**coefficient**	−1.207	0.0045698	−5.7832×10^−6^	2.437×10^−9^
**error**	0.12433	0.0004144	4.5905×10^−7^	1.691×10^−10^

R = 0.99836; χ^2^ = 1.5935×10^−5^.

If each point of the curve of Fig. 2 were a point taken at constant temperature and pressure, the enthalpy change of the transformation, 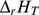, would be given by:
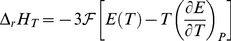
(3)The temperature dependency of eq 3 should be written as given in any chemical thermodynamics textbook by equation:
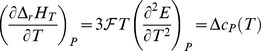
(4)being 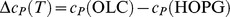 and 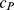 the molar heat capacity at constant pressure. In the case of reaction B and at the experimental temperatures, it is expected that 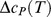 should be quite low. Equation 4 implies the same value of pressure at each temperature for both the electrodes, i.e., 
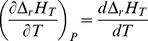
. The 1^st^ partial derivative against *T* of eq 3, i.e. eq 4, shows too high 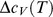 values which are unexpected for the process under study. For this reason, the assumption of constant pressure is not more valid. Therefore, the total derivative of 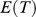, i.e.,
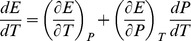
(5)should be considered where,
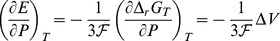
(6)being 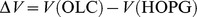 and 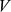 is the molar volume. By substituting eq 6 in eq 5 and combining with eq 3, the *P*, *T* dependency of 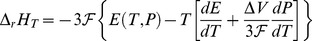 is found:



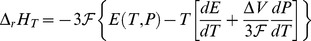
(7)Accordingly, eq 7 differs from eq 3 for the extra term 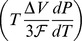, which entails 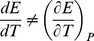.

The 1^st^ derivative of eq 7 with respect to *T* is:
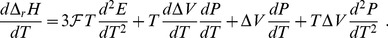
(8)From the total differential of 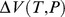, the relationship below follows:
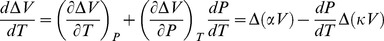
(9)where α and κ are, respectively, the isobaric volume expansion coefficient and isothermal compressibility. By making use of eq 9, eq 8 can be conveniently modified in the form:
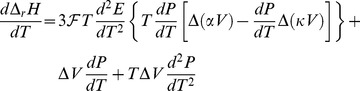
(10)
Equation 10 should be compared with well-known equation [17]:
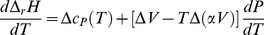
(11)which states that if some phase transition occurs, the equilibrium pressure does not remain constant as the temperature is varied. Since we can write that 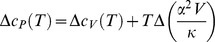, by equating eq 10 and eq 11, the following 2^nd^ order differential equation for *P* is found:



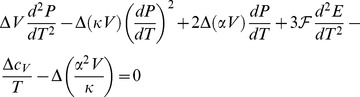
(12)


**Figure 3:**
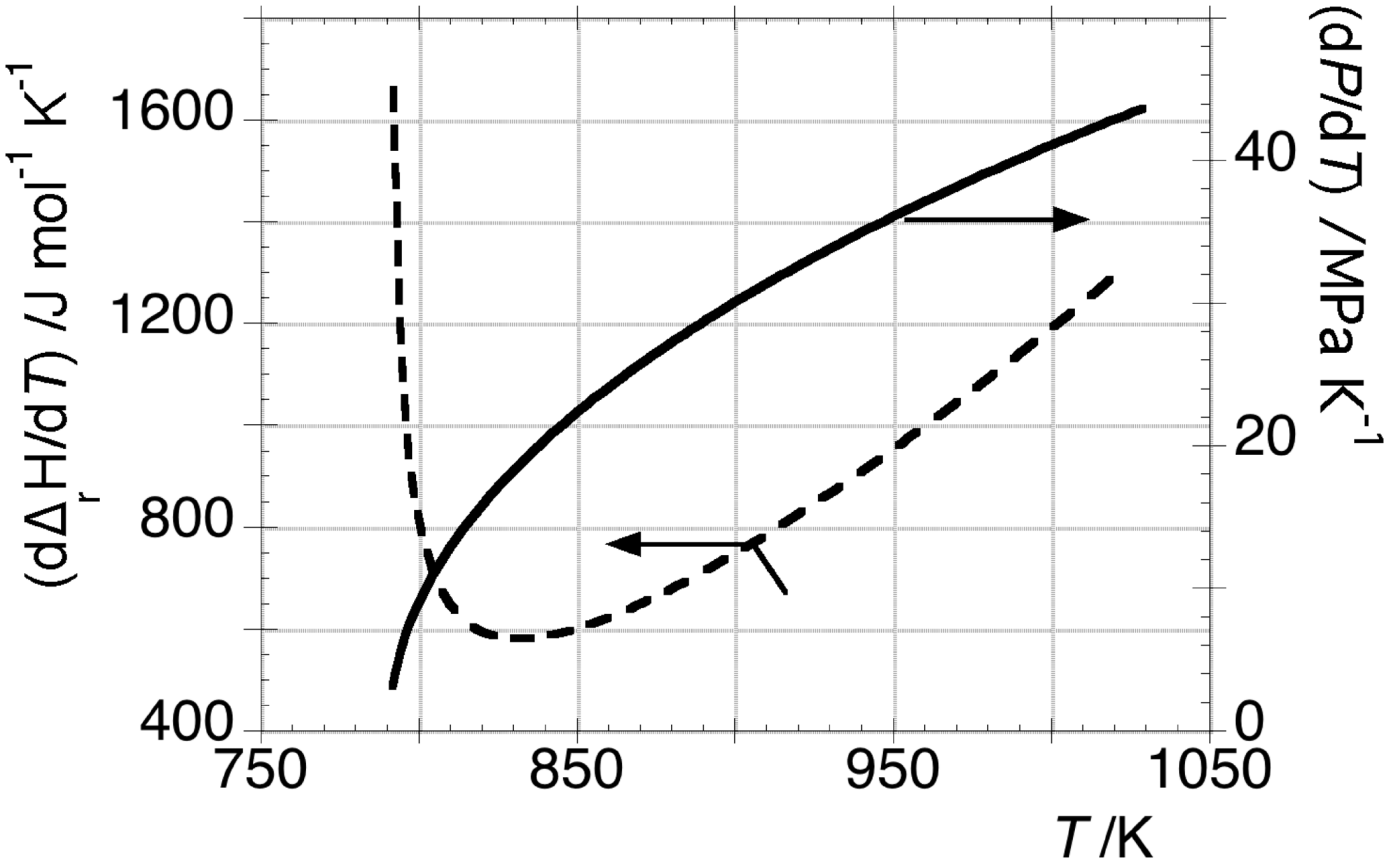
Representation of 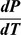 vs *T* obtained by solving eq 13 (right axis) and trend of 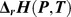 calculated by eq 11 (left axis).

To simplify eq 12, we can reasonably neglect 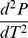 and assume 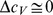 being both OLC and HOPG graphitic materials at the same quite high temperature where 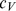 is for both ones close to 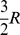. Therefore, eq 12 becomes a quadratic equation in the 1^st^ derivative with respect to *T* of the pressure:



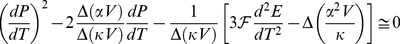
(13)The solution of eq 13 has been obtained by using the experimental data, 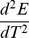, and the knowledge of the *T* and *P* functions of the physical data of HOPG and OLC. The *T* functions are reported in Table 2 and their related coefficients given in Table 3. Unfortunately, α and κ data of OLC are unavailable and we utilized, as first approximation, the data of fullerene C_60_ [18]. Due to lack of data on the temperature dependency of the OLC molar volume, an estimation can be done starting from the definition of the isobaric expansion coefficient 
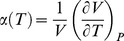
 and its *T* dependency reported in Table 2 together the coefficients for C_60_ given in Table 3. Therefore, equation below has been considered:
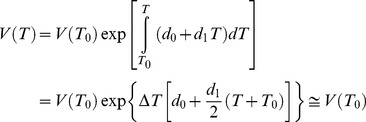
(14)where 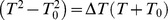 holds. The final approximated value is justified by the amount in braces, which is 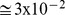 being 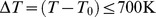. The values of 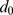 and 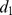 are reported in Table 3. The 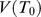 value was taken equal to 7.31×10^−6^ m^3^ mol^−1^ at 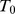 = 298 K which is the 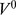 value of OLC [19]) (see Table 3). Due to the lack of data on the pressure dependence of *α*, *κ* and 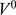 for OLC, all these parameters were considered constant with pressure for the carbon species present in both electrodes. This choice was mandatory to ensure a balanced behaviour to both OLC and HOPG. Figure 3 shows the trend of 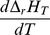 vs *T* in the left axis calculated according to eq 11 after solving eq 13 to find 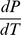, being the latter quantity plotted on the right axis. It should be noticed that the shape of the curve 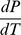 might be affected by the approximation 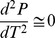 introduced in equation 12.

**Table 2:** Temperature functions of the molar volume, 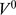, isobaric volume expansion coefficient, *α*, and isothermal compressibility, *κ*, used for computing eq 13.

	*V* ^0^(*T*)[a]	*α*(*T*)[b]	*κ*(*T*)[b]
**HOPG^25^**	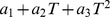	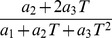	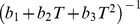
**OLC**	*V* ^0^	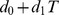	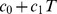

[a] Fullerene C_60_ data were assumed for OLC from reference [18] (see also text) with the exception of 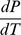, which is known [19].

[b] Linear equations for OLC (C_60_) were taken up from the plots in reference [18].

**Table 3:** Values of the coefficients of Table 2.

Coefficient [a]	Value	Ref.
***a*** **_1_/m^3^mol^−1^**	5.30×10^−6^	[25]
***a*** **_2_/m^3^ mol^−1^K^−1^**	2.14×10^−10^	[25]
***a*** **_3_/m^3^ mol^−1^K^−2^**	1.95×10^−14^	[25]
***b*** **_1_/Pa**	3.63×10^10^	[25]
***b*** **_2_/Pa** **K^−1^**	−1.32×10^7^	[25]
***b*** **_3_/Pa K^−2^**	9.45×10^3^	[25]
***c*** **_0_/Pa^−1^**	8.37×10^−11^	[18]
***c*** **_1_/Pa^−1^K^−1^**	7.66×10^−15^	[18]
***d*** **_0_/K^−1^**	4.30×10^−5^	[18]
***d*** **_1_/K^−2^**	4.27×10^−9^	[18]
***V*** **^0^/m^3^mol^−1^**	7.31×10^−6^	[19]

[a] The original values of coefficients 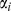 are given in nm^3^. They were multiplied by 10^−27^ *N*_A_ being *N*_A_ the Avogadro number.

**Figure 4:**
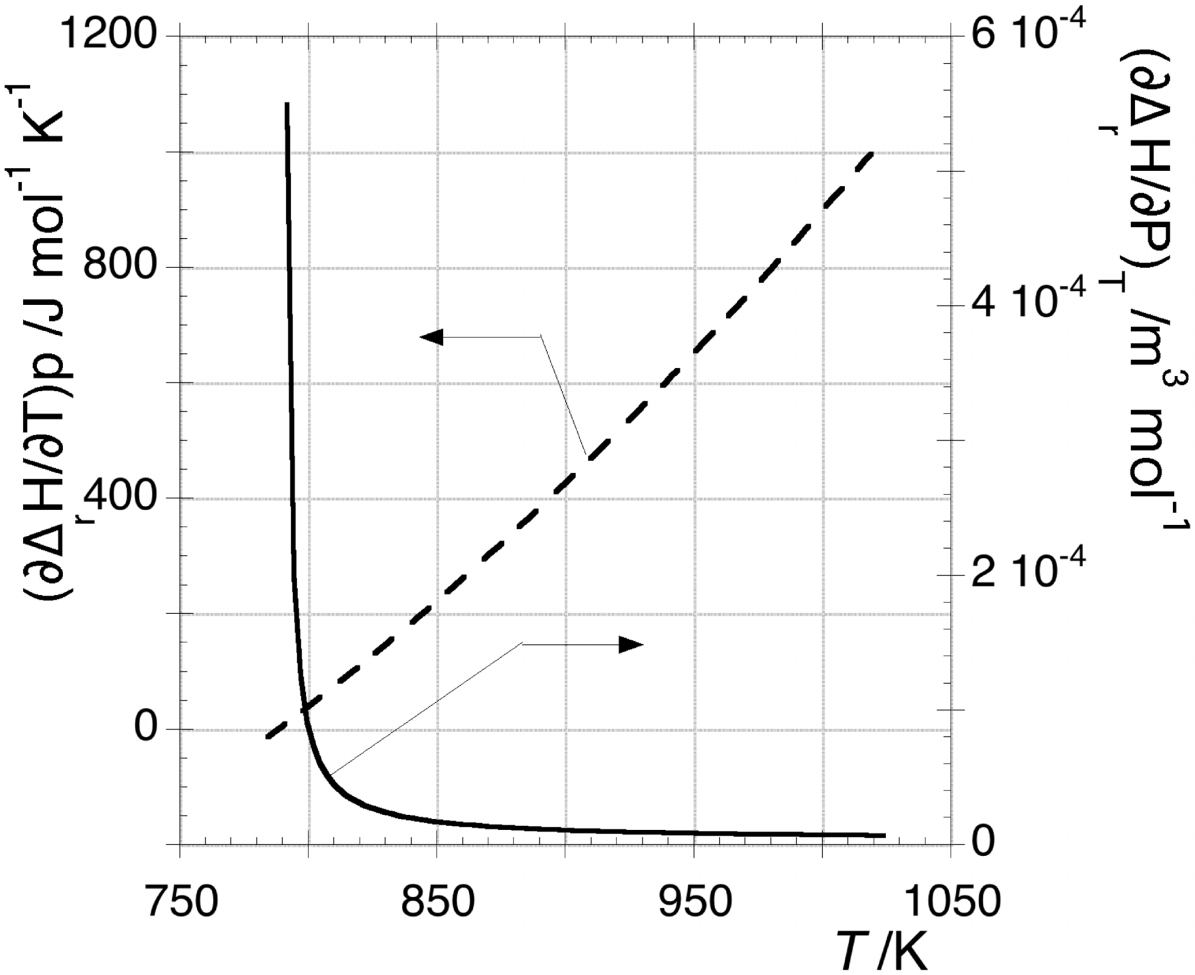
Partial derivatives of 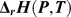. The quantity in the left axis was calculated according to eq 4. The quantity in the right axis was calculated by the total differential of 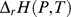 through 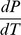 vs *T* function, obtained by solving eq 13.

From the total differential of 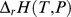 the quantity 
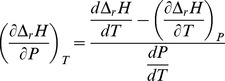
 can be obtained and plotted against *T* in Fig. 4 (right axis) together 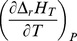 (left axis). Equation 4 was used for this calculation.

**Figure 5:**
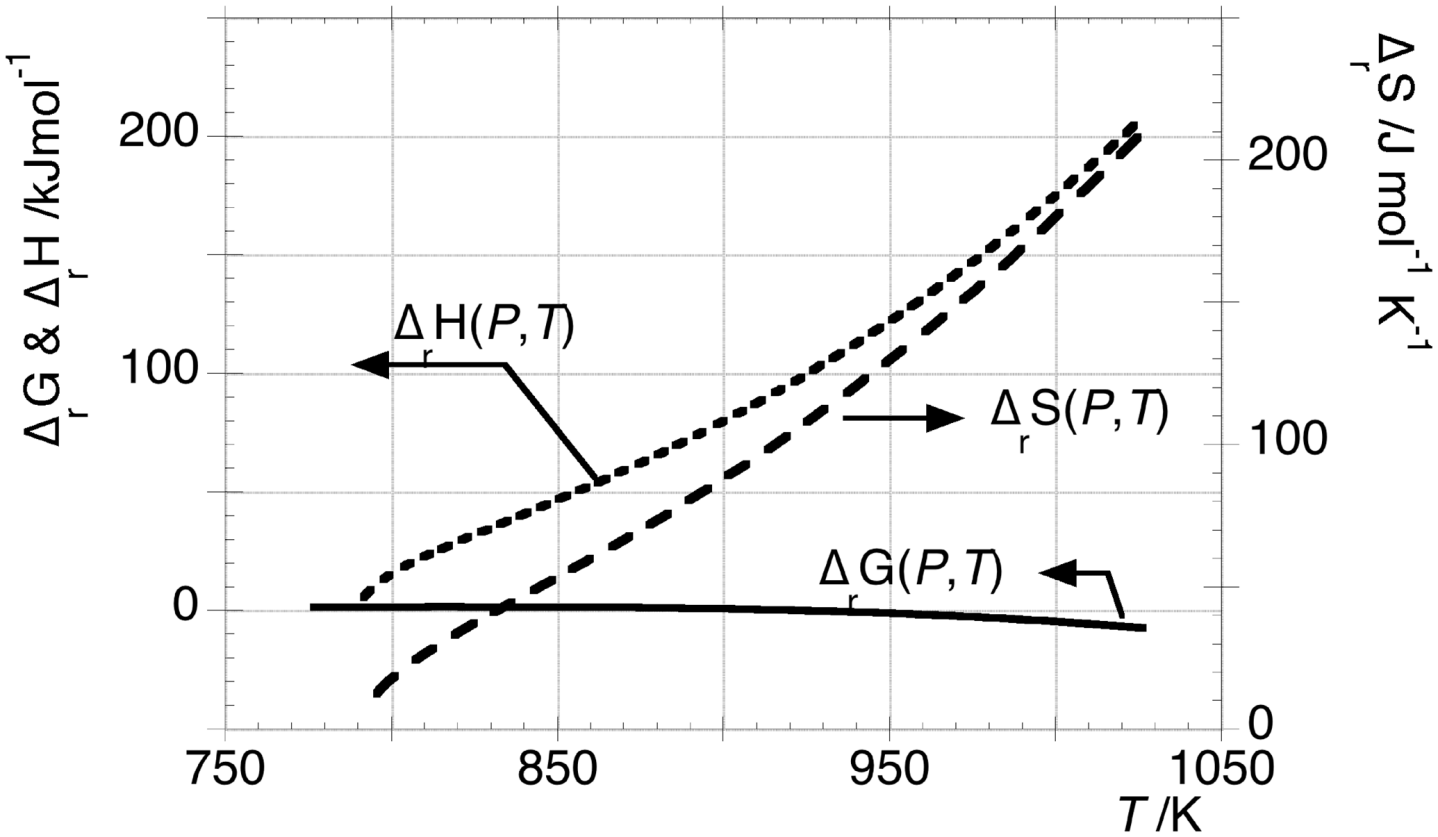
Representation on left axis of 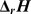 and 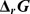 vs *T* obtained respectively by eq 7, after solving quadratic equation 13, and eq 1. On the right axis, the trend of 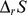 calculated by eq 15 is also displayed.

The entropy change of transformation B as function of temperature and pressure, was calculated as:



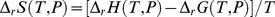
(15).This equation can be calculated through eq 7, after solving the quadratic eq 13, by using the experimental data of 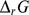 reported on Fig. 2. These thermodynamic functions are shown in Fig. 5. At 920.6 K, 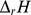 and 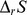 values are 95.8 kJ mol^−1^ and 104.1 JK^−1^ mol^−1^, respectively.

**Figure 6:**
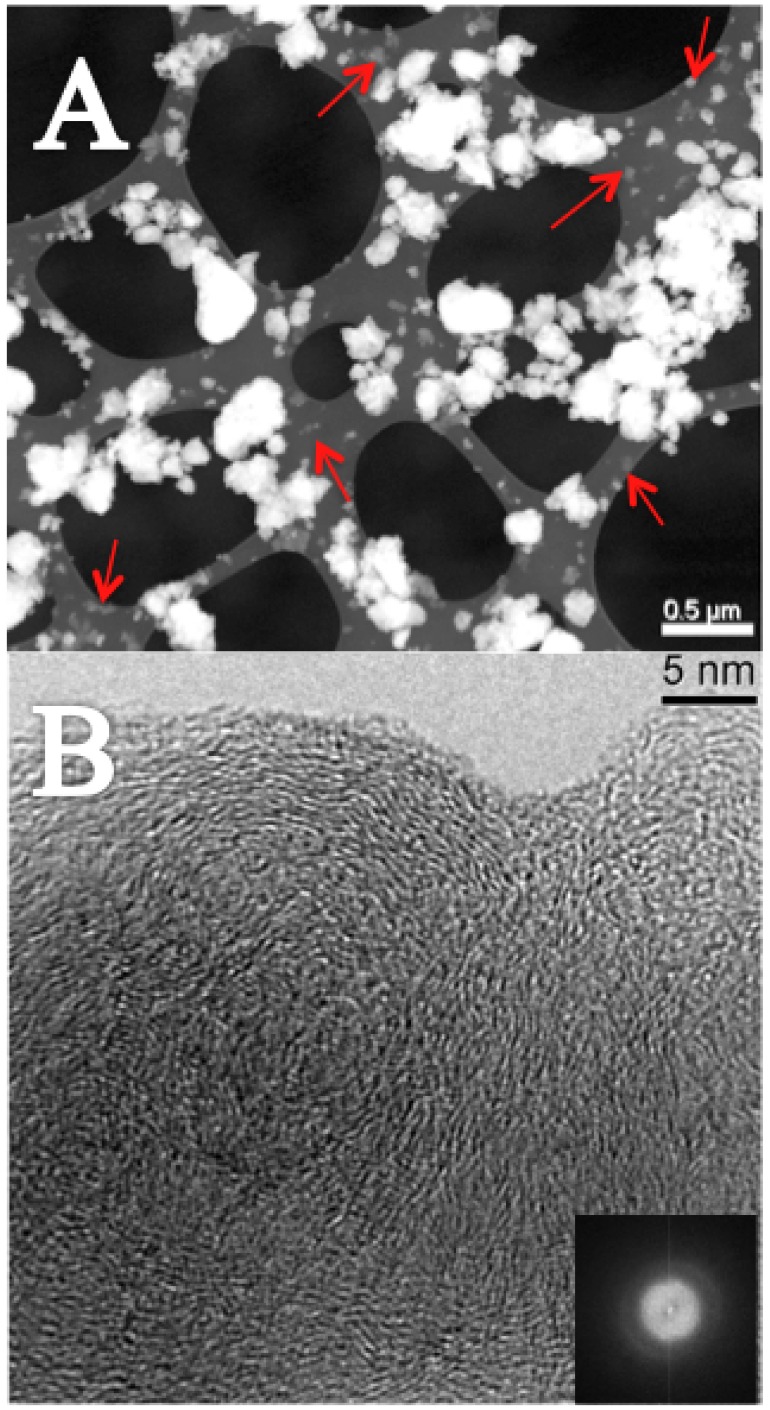
Before the experiment. Panel A: STEM image of the powder of the electrode containing OLC. The OLC nanostructures appear on the holey carbon grid as spot with very light contrast. Some of them are indicated with red arrows. Panel B: HR-TEM of OLC. The FFT image in the inset shows poor crystallinity of OLC.

Why does the pressure change with temperature in our experiment? The experiment is carried out under high vacuum and the static pressure on the cell is maintained constant at about 0.1 bar as described in the experimental section (see § 1.3 of the Supporting Information S1). Thus, the present experimental conditions cannot justify the presence of the 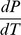 quantity. The explanation for 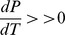 should be searched in the growth of the pressure inside the cell, which is caused by a process occurring under volume constraint. The positive sign of *emf* indicates that 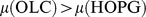 where *µ* is the chemical potential of carbon. By itself this does not justify the increase of *P* with *T* but the trend of *emf* with *T* suggests it. Furthermore, there are experimental evidences indicating that the electrode containing OLC is the site where the volume constraint is particularly high. They arise from the comparative analysis of both electrode powder mixtures, before and after the experiments, by making use of x-ray diffraction (XRD), micro Raman spectroscopy (mR) and high-resolution transmission electron microscopy (HR-TEM):

- The XRD patterns spectra demonstrate that no chemical change occurred (see §1.5.1 of the Supporting Information S1). The large increase of crystallinity of CrF_2_ was the unique difference found in XRD. Due to their almost amorphous state, OLC give low intensity XRD features differently from HOPG, which is very well revealed. In the present case, the situation is complicated by the presence of features of other species in the electrode that superimpose and/or they are very close to the OLC features (see §1.1.2.2 of the Supporting Information S1). It is reported [20] in high temperature – high pressure experiments on OLC, that their most intense feature (*d*_200_ = 0.354 nm, 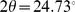; see §1.1.2.2 of the Supporting Information S1) is negligibly shifted as function of pressure at constant *T*. For instance, at 500 °C the lattice parameter changes with pressure as −9.6×10^-13^ nmPa^−1^. At our calculated pressures, which are in the order of tens of MPa (see below), a shift of 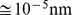 is expected, which is clearly undetectable. The mR spectroscopy does not reveal differences (see §1.5.2 of the Supporting Information S1) with exception of some changes in the relative intensities, which are meaningless. There is superimposition of the OLC and HOPG features with the features of the mixture Cr_3_C_2_ - CrF_2_ (see §1.1.2.3 of the Supporting Information S1). Therefore, though this inconvenient, the absence of any new feature is a proof that both carbon species remain unchanged;
- HR-TEM and STEM analyses show a very different scenario of the electrode powder with OLC before and after the experiments. Before, OLC are easy identifiable in the mixture as reported in the STEM image of Fig. 6A. Free OLC nanostructures (red arrows) are quite abundant, where they can be seen either as spots with very light contrast on the holey carbon grid or mixed with the Cr_3_C_2_-CrF_2_ powder. The HAADF-STEM mode is also known as Z contrast mode because the intensity in each point of the image is proportional to 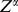 with 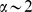 and *Z* the atomic number. Carbon appears dark, as it is much lighter than the other compounds present in these samples. The OLC morphology at the nanoscale is shown in Fig. 6B and is found in agreement with previous experimental results [7] [21]. The inset in Fig. 6B shows the Fast Fourier Transform (FFT) of OLC where the reflections of graphitic planes appear quite diffuse.

**Figure 7:**
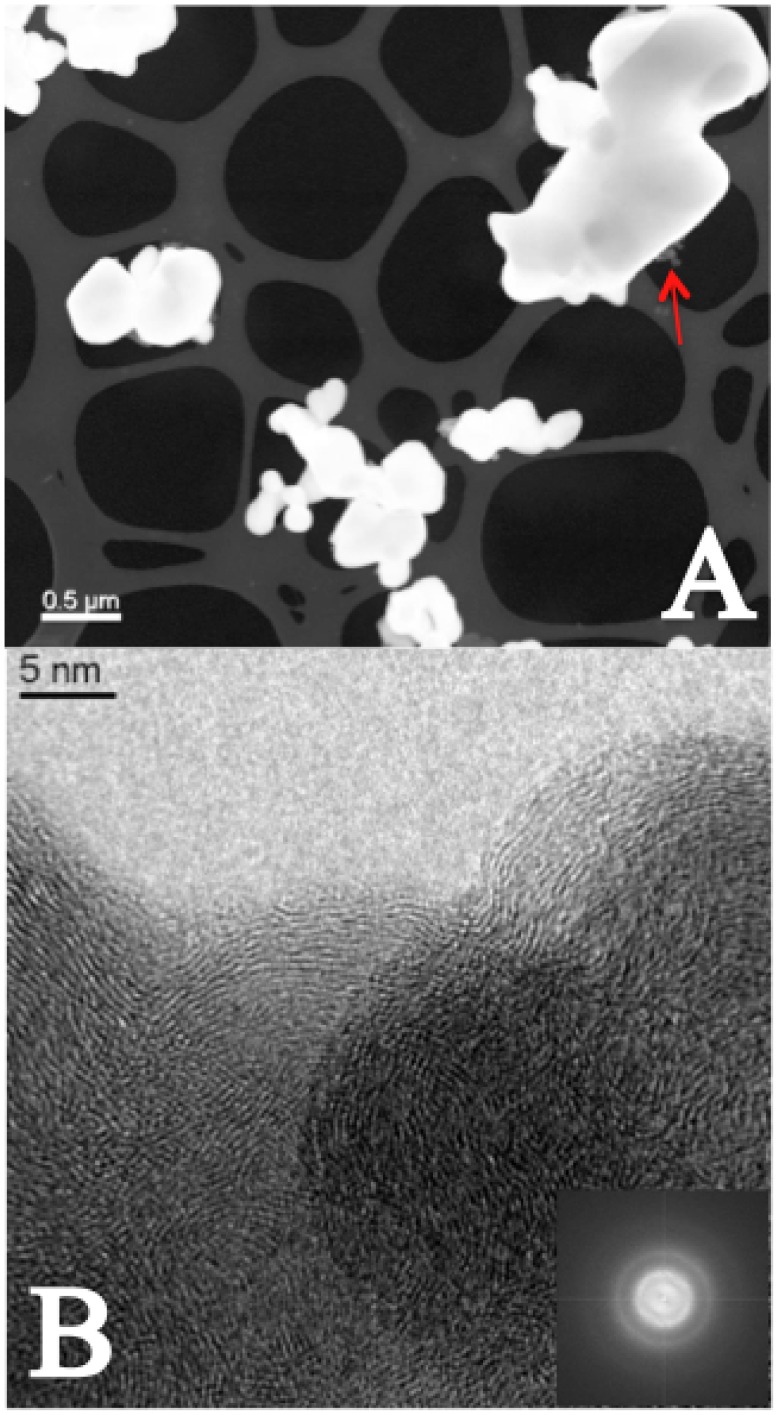
After the experiment. Panel A: STEM image of the powder of the electrode containing OLC. The OLC nanostructures are practically disappeared. The holey carbon grid is clean and only large grains with rounded shape stay on the grid. A few visible OLC are indicated with red arrow. Panel B: HR-TEM of OLC. The FFT image in the inset shows that the crystallinity of OLC is higher with respect to the FFT image in Figure 6B.

The powder mixture of the same electrode after the experiment appears completely different: OLC are practically disappeared while large grains with round shaped contours were found as shown in the STEM image in Fig. 7A. Free OLC are hardly observable: just small pieces on the agglomerates, while the holey carbon grid is practically clean. The analysis on the very few OLC found in the sample reveals some significant differences with respect to the OLC before the experiment: the graphitic planes form now better ordered quasi-spherical structures (see Fig.7B). The electron energy loss spectroscopy measurements (see § 1.5.3. of the Supporting Information S1) agree with this result: the sp^2^ carbon coordination values were found 82±3% and 86±3% for OLC before and after, respectively and the result seems to point to a further graphitization of OLC towards multishell ordered structures in the process (see discussion below). The sintered agglomerates usually are so thick to result non-transparent to electrons and to prevent the analysis of their internal structure. When small particles are found, the EELS revealed typical features of variable size inside the particle. Their contrast is typical of cavities, but it was not possible to assess whether they are empty or possibly filled with OLC. This is the most reasonable hypothesis, being OLC otherwise missing.

**Figure 8:**
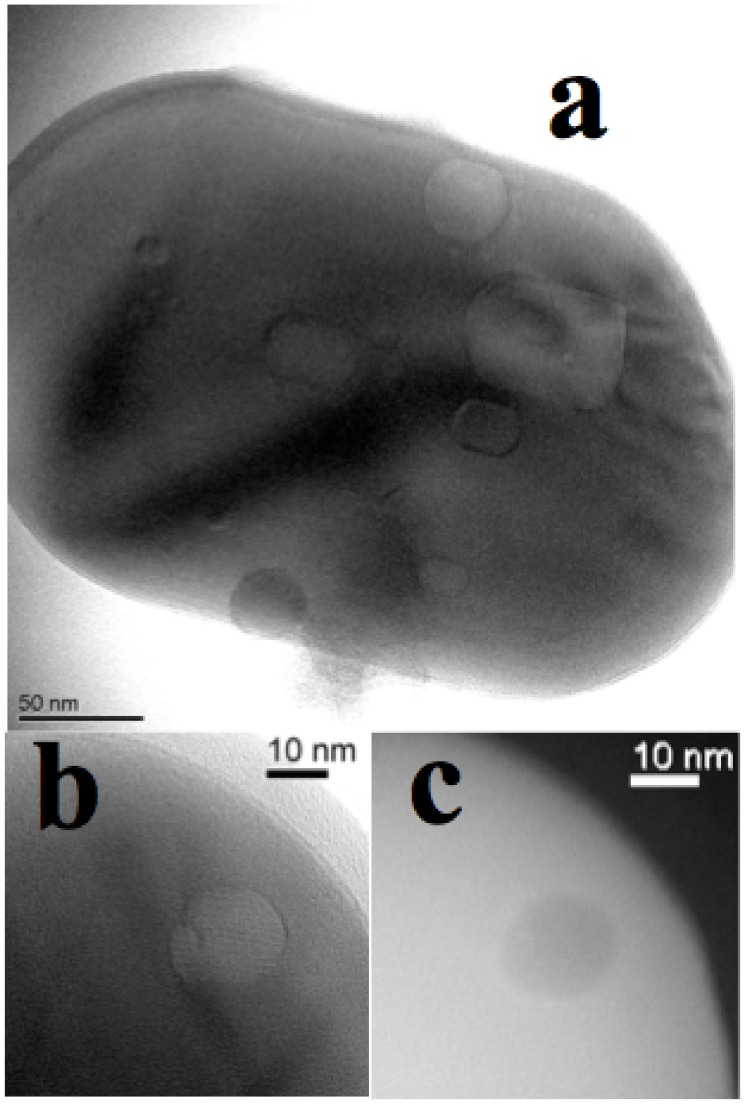
Zero loss TEM image (Panel a) of a typical small sintered particle (as the particles in Figure 7A) of the electrode with OLC after the experiment. The particle contains several cavities, that can be empty, or at least full of material much lighter than the surrounding sample, as it is proven by the comparison between the HRTEM (panel **b**) and STEM (panel **c**) images of the same cavity, that is whiter in **b** and darker in **c**.

The zero loss TEM image of a typical small sintered particle containing several cavities is shown in Fig. 8a. The fact that these cavities are empty or filled with material lighter than the surrounding sample is proven by the comparison between the HRTEM and STEM images in Fig. 8b and Fig. 8c of the same cavity, that is whiter in b and darker in c.

**Figure 9:**
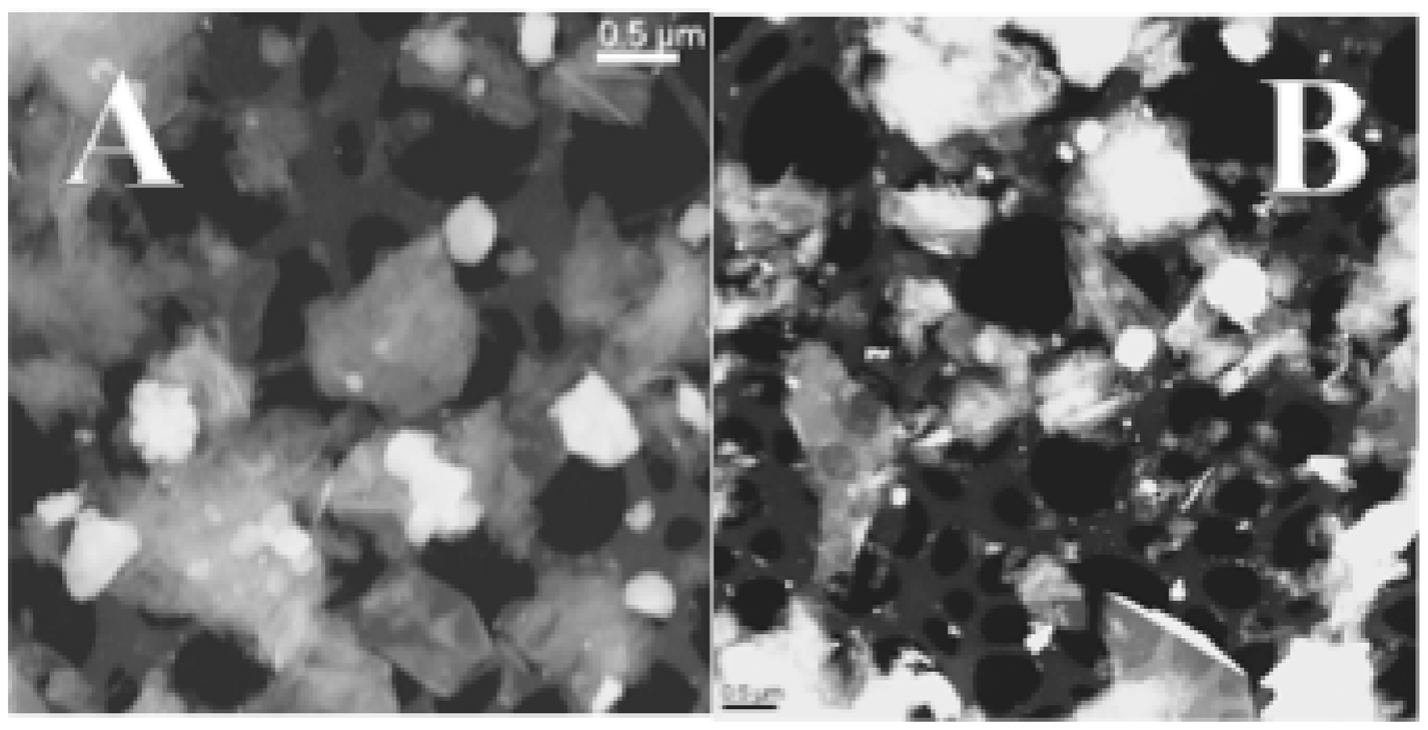
STEM images of the electrode containing HOPG. Panel A, **before the experiment**. Panel B, **after the experiment**. No differences were found and no sintering was observed.

The electrode containing HOPG and the same Cr_3_C_2_ - CrF_2_ mixture does not show any modification after the experiment, as it can be seen by comparing the STEM images in Fig.9 A and B.

The following considerations arise from the above evidences:

- The temperature range explored in the experiments does not allow suspecting any vaporization of carbon from OLC. The C-nanostructures are still in the electrode;
- High temperatures and experiments lasting hundreds of hours create the ideal conditions to favour the sintering of the whole mixture where OLC remain embedded in the very hard and refractory structure of Cr_3_C_2_. The melting points of Cr_3_C_2_ and CrF_2_ are respectively 2168 and 1167 K. The highest temperature reached in the experiments was 1030 K, which is close to the melting point of CrF_2._ Thus, it is expected that its softening acts as a sintering medium of the whole mixture. The high specific surface area of OLC (>100 m^2^g^−1^, see § 1.1.1 of the Supporting Information S1) contributes to this process and facilitates the embedding of the nanostructure. Therefore, the role of OLC nanoparticles seems to be crucial and it explains the absence of any modification of the electrode containing HOPG (and the same Cr_3_C_2_-CrF_2_ mixture) which worked in the same experimental conditions;
- Consider as rough approximation the electrodes as cylinders free of expanding with temperature filled with rigid particles (sintered Cr_3_C_2_-CrF_2_ mixture) embedding nanoparticles (OLC) the dilatation of which is hindered. By imposing no volume change for both OLC and HOPG, the respective 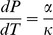 values can be evaluated and compared as follows:

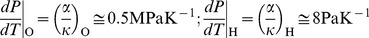
(16)The subscripts O and H stand for OLC and HOPG. The temperature dependence of the quantities entering eq 16 has been neglected in the computation and the coefficients *a*_2_, *b*_1_, *c*_0_ and *d*_0_ were only retained in the expression reported in Table 3. The values found in eq 16 demonstrate clearly the great difference in the internal pressures to which both carbon forms are subjected in the respective electrodes. For the sake of comparison, the quantity 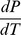 for Cr_3_C_2_ is: 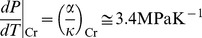 where *κ* [22] and *α* [23] are available in literature. Due to the negligible changes with temperature of the internal pressure (see eq 16), the chemical potential of carbon in HOPG, 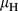, is only a function of *T* differently to the chemical potential of carbon in OLC, 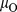, that is a function of *T* and *P*(*T*). Therefore, we can write that:
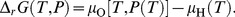
(17)


### Evaluation of the surface contribution

Owing to the nanometric size of the carbon particles, the surface contribution to the energetics of the process cannot be neglected, in principle. In the following of this section the shape of the nanoparticle is assumed to be spherical. Let us estimate the chemical potential difference using the thermodynamic approach, based on the Gibbs model for interface [24], in terms of bulk chemical potential, surface free energy and surface tension.

The difference between surface energy and surface tension plays a fundamental role when dealing with interphases in the solid state. In this respect, we briefly recall the definition of these quantities, which are needed for the discussion that follows. The surface energy excess, σ, is the reversible work required to create a unit surface at constant volume, temperature, pressure and composition [24]. In such a process the surface is not stretched during its formation. On the other hand, the surface tension, γ, is defined as the reversible work (per unit area) required to change the area of the surface through a stretching process. It is worth noticing that in this definition the infinitesimal deformation of the surface involves an “initial” configuration of the system where the solid is not stressed. By including bulk and surface contributions, the general expression of the chemical potential difference reads (see §2 of the Supporting Information S1)
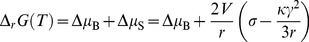
(18)where *r* is the radius of the nanoparticle and 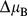 denotes the "bulk" contribution, that is 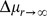. In the following we focus our attention on the impact of the surface term (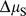) on 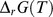 and on its temperature dependence.

The contribution of the surface tension containing term to 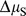 is usually smaller than that due to the surface excess free energy, 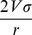. Consequently, 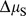 is expected to be higher than zero. Typical values of the quantities entering eq 18 are [25] *V* = 5.36×10^−6^ m^3^ mol^−1^, κ = 3.24×10^−10^ Pa^−1^ and *r* = 10 nm. As far as the *σ* value is concerned, for nanocarbon structures, such as fullerene, single and multi walled nanotubes, it is found [5] to range between 0 and 0.045 Jm^−2^. On this basis, the upper bound of 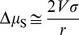 is estimated to be about 50 Jmol^−1^ for *r* = 10 nm.

As far as the temperature dependence of 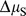 is concerned, it is ascribed to the temperature dependence of the physical quantities entering in eq 18. In particular, the temperature dependence of *σ* is given by the Gibbs adsorption equation that, for one component system, implies 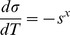, where 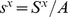 is the excess surface entropy. From this equation, neglecting as a first approximation the temperature dependence of the excess entropy in the temperature range here considered, we get 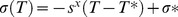 where T* is the temperature at which 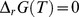. Concerning the temperature dependence of the surface tension, given by eq 1 in the Supporting Information S1 (§ 2), it is obtained
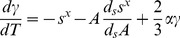
(19)where we exploit the fact that the surface energy is a function of the independent variables temperature and “stretched area” *A* (the subscript *s* stands just to remind us that the variation is performed by stretching). According to the discussion that follows this derivative can be estimated using the Born-Stern method [26]. In this approach, the entropy excess per unitary area of a solid-vacuum interface is equal to 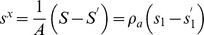 where *S* is the entropy of the actual solid, *S'* the entropy of the model system (without interface), *ρ_a_* the surface density and 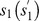 the entropy, per atom, of surface (bulk) species. By assuming, as usual, 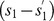
independent of temperature and deformation of the surface 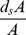, for 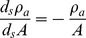 one obtains 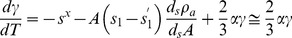. For instance, by considering the excess entropy equal to the change in the vibrational entropy of the atom the expression 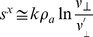 holds where 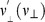 is the vibrational frequency of the surface (fully coordinated) atom along the “broken bond” direction. Since for the number of mole of atoms in the particle, *N*, is constant we set 
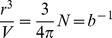
 in eq 18 to obtain 
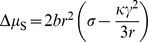
. Moreover, 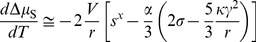
(20)where α is of the order of 10^−6^ K^−1^. A crude estimate of the vibrational contribution of 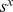, on the basis of the Kossell model [26] shows that 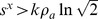 that implies, using 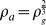 with *ρ_g_* the graphite density, a value of 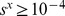 J m^−2^ K^−1^. This figure is about two orders of magnitude larger than α. Moreover, 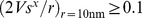 J mol^−1^ K^−1^. From eq 15, the value of 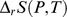, in the middle of temperature range, is equal to 96.75 Jmol^−1^K^−1^, which is not consistent with the value 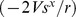.

The present analysis indicates that the bulk term is the dominant contribution both to the chemical potential change of transformation B and its derivative on *T*. At this point, a comment is in order on the use of the Gibbs model for dealing with OLC systems. In fact, in this approach “bulk” and surface terms sum up as two independent contributions where the “size effect” is usually contained in the surface term only, i.e., 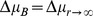 is independent of *r*. On the other hand - still remaining in the framework of the Gibbs model - owing to the variable curvature of the sheets, which made up the nanoparticles, in the OLC the “bulk” term has to be considered a function of the particle radius. For this reason in the following we refer to “atomistic” approaches for determining the energetics of the OLC particles. In fact, in these methods a suitable interaction potential functions for the C atoms is used and this makes it possible to estimate 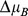 as a function of *r*.

**Figure 10:**
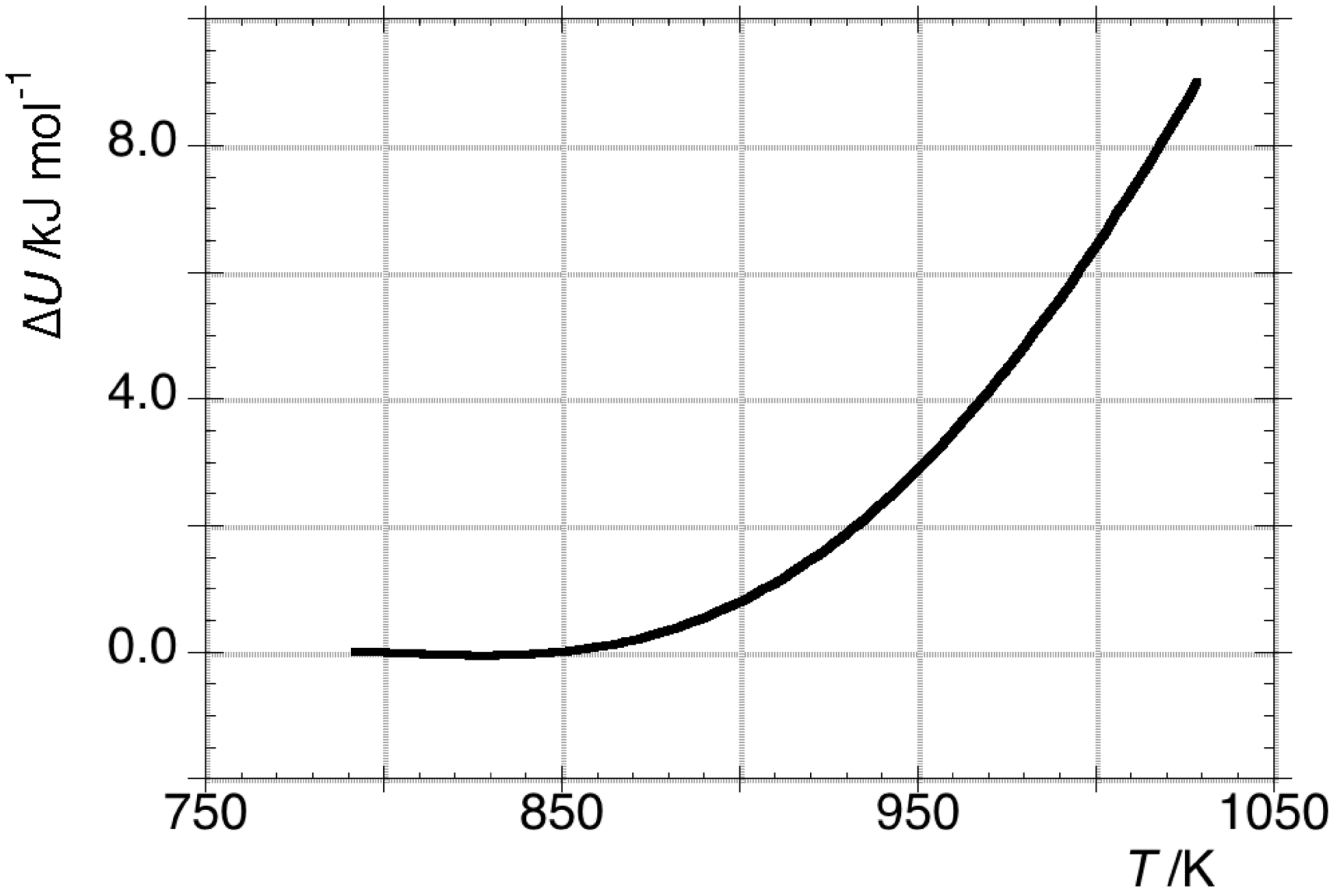
The change of internal energy 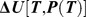 of OLC is reported against temperature according to eq 22.

Now we focus our attention on the internal energy of the particle due to the isothermal transformation from a relaxed state at internal energy *U*_0_ and volume *V*_0_ to a stressed state at *U* and *V*. To do this the *energy equation*, namely 
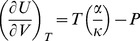
(21)was utilized. The integration of eq 21 gives the change of internal energy as (see §3 of the Supporting Information S1):



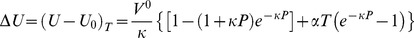
(22)The value of *V* ^0^, *α* and *κ*κ are given in Tables 2 and 3. The pressure as function of *T* was obtained by numerical integration of 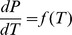 assuming *P* = 0 at *T* = 776.6 K, which was the lowest experimental temperature. The resulting function satisfies the relationship 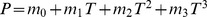
where *m*_0_ = 1.2947×10^6^ bar; *m*_1_ = −4187.2 bar K^−1^; *m*_2_ = 4.2571 bar K^−2^; *m*_3_ = −1.3067×10^−3^ bar K^−3^ with correlation coefficient 0.9999. At the highest temperature, the maximum pressure value is about 70 kbar. The trend of 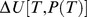 with temperature, which is almost perfectly quadratic, is plotted in Fig. 10.

It is worth noticing that the 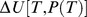 values and its trend with temperature are not comparable with values and trend of 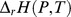 reported in Fig. 5. For example, at 903 K, which is the temperature in the middle of the explored temperature interval, the ratio 
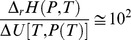
.

Due to such a high ratio jointly to the very small surface contribution, we assume that as a consequence of the high internal pressure another process connected to B should be considered. We are likely dealing with a reversible process that requires the formation of C defects in OLC, probably already defective. Thus, the whole process determining the *emf* of the cell could be written as:
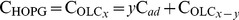
(B1)instead of process B.

**Figure 11:**
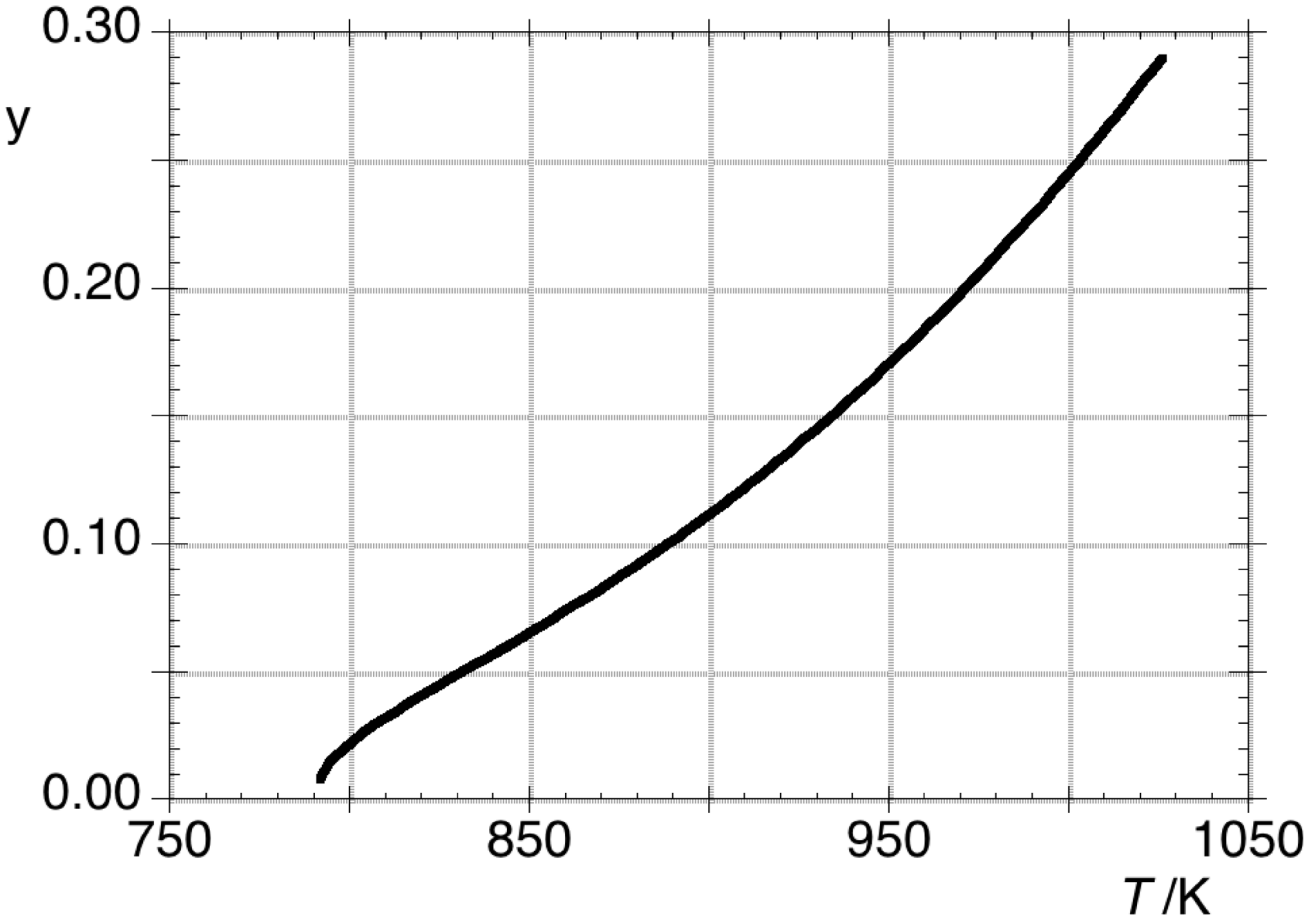
Trend of the change of the fraction of C defects in OLC against *T*. One assumes that the real transformation involves equilibrium 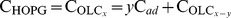 and *y* is calculated by eq 23.

Obviously, this implies that the previously considered relation 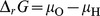 should be written as 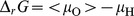 where 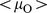 is the mean chemical potential of carbon atoms in the electrode containing the OLC nanostructures. In this quantity, the C atoms formed should be considered adsorbed atoms, C*_ad_* the formation of which causes dangling bonds. A rough evaluation of the defect fraction in equilibrium B1 can be evaluated through equation:
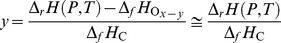
(23)where 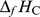 and 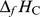 are the enthalpy of formation per carbon atom of defective OLC and free carbon atom (7.43 eV at 298 K) [27], respectively. The quantity 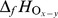 depends on the number of shells as described for multishell fullerene (MSF) made of concentric circumspheres of polyhedra *C_n_* (*I_h_*, *n* = 60*k* ^2^) and *C_n_* (*I_h_*, *n* = 180*k* ^2^) where *k* = 1,2 up to *L* is any positive integer representing the number of shells. *I_h_* and *n* are respectively the symmetry group and number of C atoms. According to the literature [28], the quantity 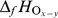 is of the order of magnitude of meV and therefore it is negligible when compared to 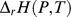 which is about 1 eV. Even the formation energy of a single carbon shell starting from graphene implies negligible energy values ranging from 331 to 34 meV at^−1^ for C_60_ and C_960_, respectively (see J. Bernholc et al. [3]). As stated in the above reference, the authors showed that the larger the size of the shell, the lower the energy of formation. Figure 11 shows how *y* changes with *T*.

Due to the assumption for obtaining eq 23, the trend of *y* is the same as 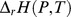 shown in Fig. 5. Anyway, the value of *y* is likely consistent with a change corresponding to one or more OLC shells. The experimental evidence through the *emf* vs *T* data shows that this process occurs reversibly. In addition, defects imply the formation of dangling bonds, which also contribute to the increase of the entropy change.

## Conclusions

In the present experimental work, we obtained high temperature data of the stability of onion-like carbon with respect to highly oriented pyrolytic graphite. Since this was performed in reversible way, through the *emf* measurement of a galvanic cell, the quantities related to the transformation investigated have to be considered reliable thermodynamic data.

The evidence that in the operating conditions the *emf* data cannot be only function of temperature allowed measuring the changes of the chemical potential of carbon in OLC under volume constraint. The volume constraint is reasonably proven by the HR-TEM images where the rigid cages made of sintered CrF_2_–Cr_3_C_2_ mixture likely embed OLC.

The positive value of 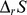 and its increase with temperature indicate that the OLC is a nanostructured system with a large number of defects. The change of their fraction with *T* was evaluated. The present analysis indicates that the bulk term is the dominant contribution to both chemical potential and entropy change of the transformation.

The absence of any chemical change in the electrodes after hundreds hours of work at high temperature guarantees the whole reliability of the experiments.

## Supporting Information

Supporting Information S1 (DOC)
