# Supplementary material for: High Temperature Stability of Onion-Like Carbon vs Highly Oriented Pyrolytic Graphite
Source: PLoS One. 2014 Aug 25;9(8):e105788. doi: 10.1371/journal.pone.0105788 (PMC4143374; doi:10.1371/journal.pone.0105788)
Supplement: Supporting Information S1 — (DOC) [file pone.0105788.s001.doc]

Supporting Information

**High Temperature Stability of Onion-like Carbon vs Highly Oriented Pyrolytic Graphite**

Alessandro Latini^1^, Massimo Tomellini^2^, Laura Lazzarini^3^, Giovanni Bertoni^3^, Delia Gazzoli^1^, Luigi Bossa^1^ and Daniele Gozzi^1^*

**LEGEND**

1. **Starting materials for the electrode preparation**
   1. HOPG, OLC, Cr_3_C_2_, CrF_2_
   2. Characterization
      1. Thermogravimetry (TG) and Differential Thermal Analysis (DTA)
      2. x-ray diffraction (XRD)
      3. microRaman (mR)
      4. High Resolution Transmission Electron Spectroscopy (HR-TEM)
      5. Electron Energy Loss Spectroscopy (EELS)
      6. X-ray Photoelectron Spectroscopy (XPS)
   3. Preparation of electrodes and cell assembly
      1. Electrodes
      2. Cell assembly
   4. Experimental apparatus for the *emf* measurements
   5. Procedure adopted for the *emf* measurements and their data acquisition
   6. Analysis of electrodes before and after experiment
      1. X-ray diffraction (XRD)
      2. microRaman spectroscopy (mR)
      3. EELS
2. **Derivation of eq 18**
3. **Derivation of eq 22**

**_____________________**

1. Dipartimento di Chimica, Università di Roma *La Sapienza*, Roma, Italy
2. Dipartimento di Scienze e Tecnologie Chimiche, Università di Roma *Tor Vergata*, Roma, Italy
3. IMEM – CNR, Parma, Italy
   1. **Starting materials for the electrode preparation**
      1. Information on HOPG, OLC, Cr_3_C_2_, CrF_2_ as received.

|  | **Purity /%** | **Size** | **Maker** |
| --- | --- | --- | --- |
| **HOPG^a^** | 99.3 | — | Momentive Performance, Materials, Inc. (Germany) |
| **OLC^b^** | ≥ 99 | < 30 nm | Sigma-Aldrich |
| **Cr_3_C_2_** | 99 | 325 mesh | Sigma-Aldrich |
| **CrF_2_** | 97 | 80 mesh | Chempur (Germany) |

^a^ ZYH grade, mosaic spread 3.5°±1.5°. Powder obtained by exfoliating a piece of 20x20x4 mm.

^b^ Prepared by laser ablation of graphite. The specific surface area is > 100 m^2^g^-1^. The as received powder was two times treated in refluxing HCl ~6 M to remove possible metallic contaminants then washed thoroughly with deionized water until neutral pH of the washings.

- - 1. Characterization

All the figures below show the characterization of all as received substances utilized in the electrodes of the galvanic cell.

- - - 1. Thermogravimetry (TG) and Differential Thermal Analysis (DTA).


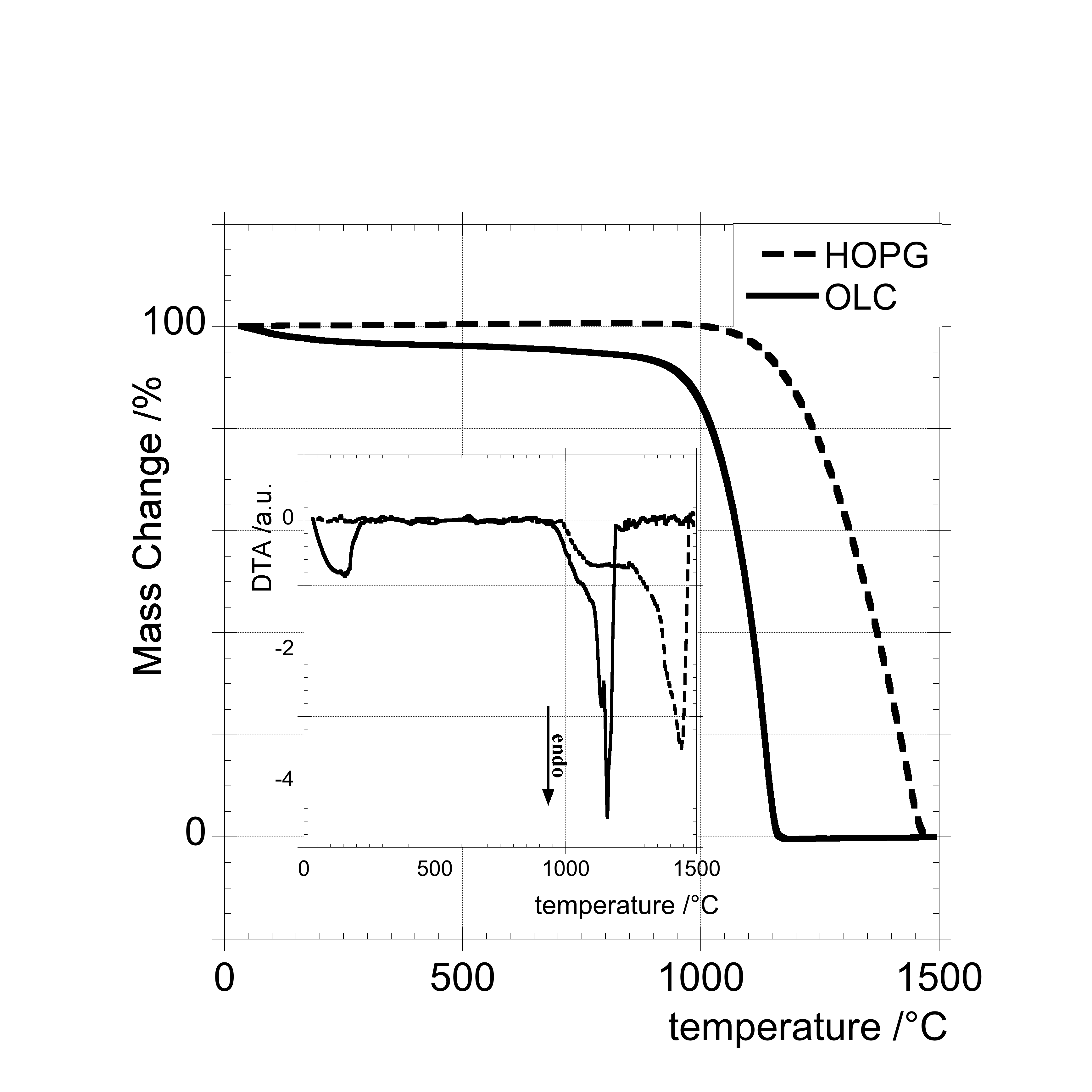
Thermogravimetric measurements were performed by a Netzsch STA 409 PC Luxx thermobalance (temperature range: RT to 1500 °C; resolution: 2 μg)

Figure S1. Thermogravimetric analysis in pure CO_2_ of ‘as received’ HOPG and OLC samples. The inset shows the respective differential thermal analyses. The purities found were 99.32 and 99.99 w% for HOPG and OLC, respectively.

in simultaneous DTA-TG mode. Temperature was measured by means of a S-type thermocouple (Pt-Pt/Rh 10%). The thermocouple was calibrated against the melting points of In, Sn, Bi, Zn, Al, Ag, Au and Ni. All the measurements were performed in alumina crucibles with a scan rate of 5 °C min^-1^, under a flow of pure CO_2_ (80 ml min^-1^ @ 1.013 bar and 21 °C). A correction measurement without sample was performed and subtracted to the TG and DTA curves of the samples. The purities found through TG for HOPG and OLC are 99.32 and 99.99 w%, respectively. The DTA curves for both materials are shown in the inset.

- - - 1.
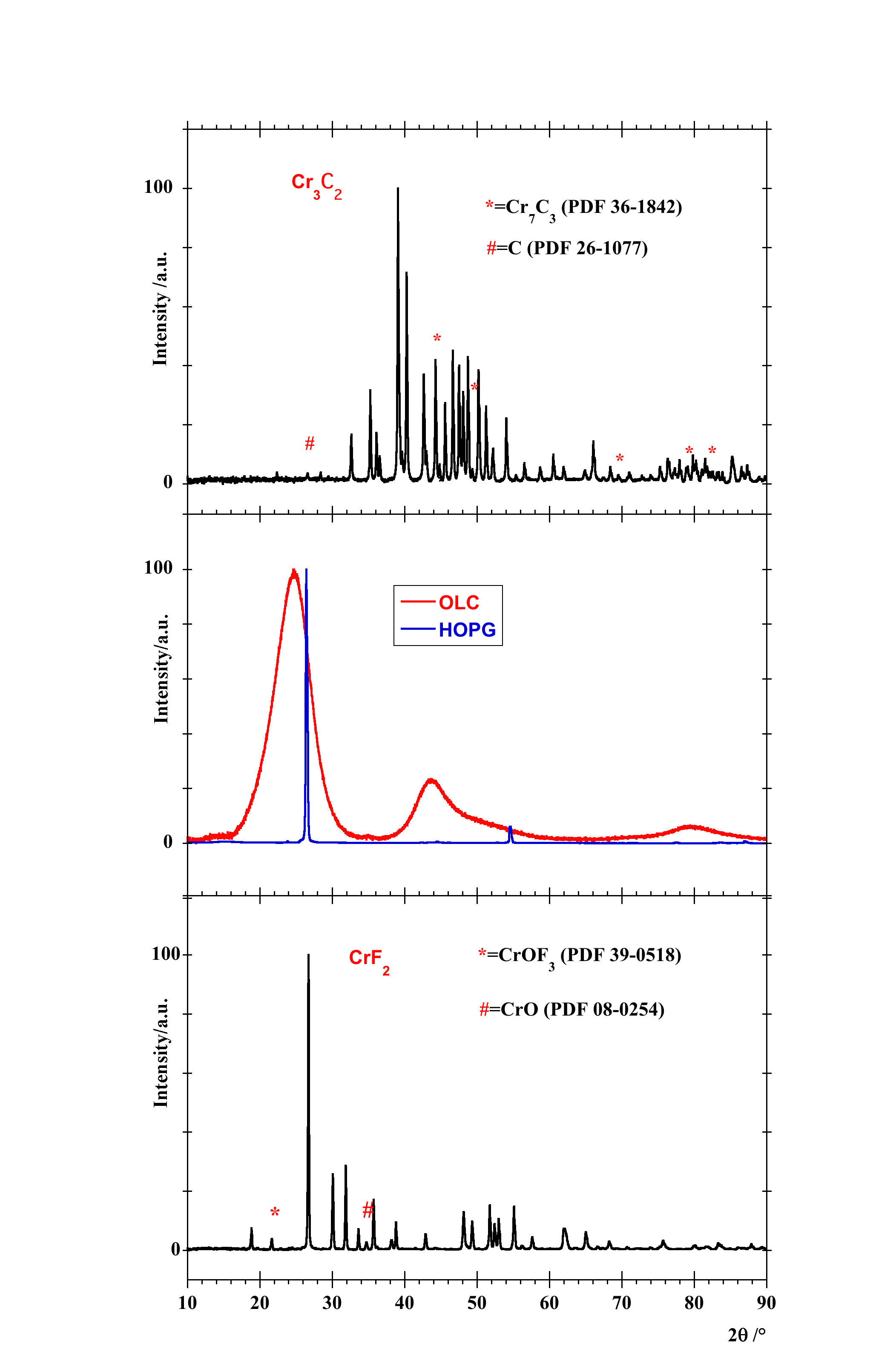
x-ray diffraction (XRD)

Figure S2. XRD spectra of all ‘as received’ compounds of the electrodes of cell A. From the top: Cr_3_C2, OLC & HOPG and CrF_2_. All the features were checked with ICDD PDF data base.

Diffraction patterns were acquired using a Panalytical X’Pert Pro diffractometer (Bragg-Brentano geometry, radiation Cu Kα, λ=0.154184 nm) and equipped with an ultrafast X’Celerator RTMS detector. The scans were performed in the 2θ range 10-90° with a resolution of 0.001°. The acquired scans were analysed using the X’Pert High Score Plus software. Making use of the ICDD PDF database, the phase identification was performed. Impurity traces were found in Cr_3_C_2_ and CrF_2_: Cr_7_C_3_ and C, chromium oxyfluoride and chromium oxide, respectively.

- - - 1. microRaman


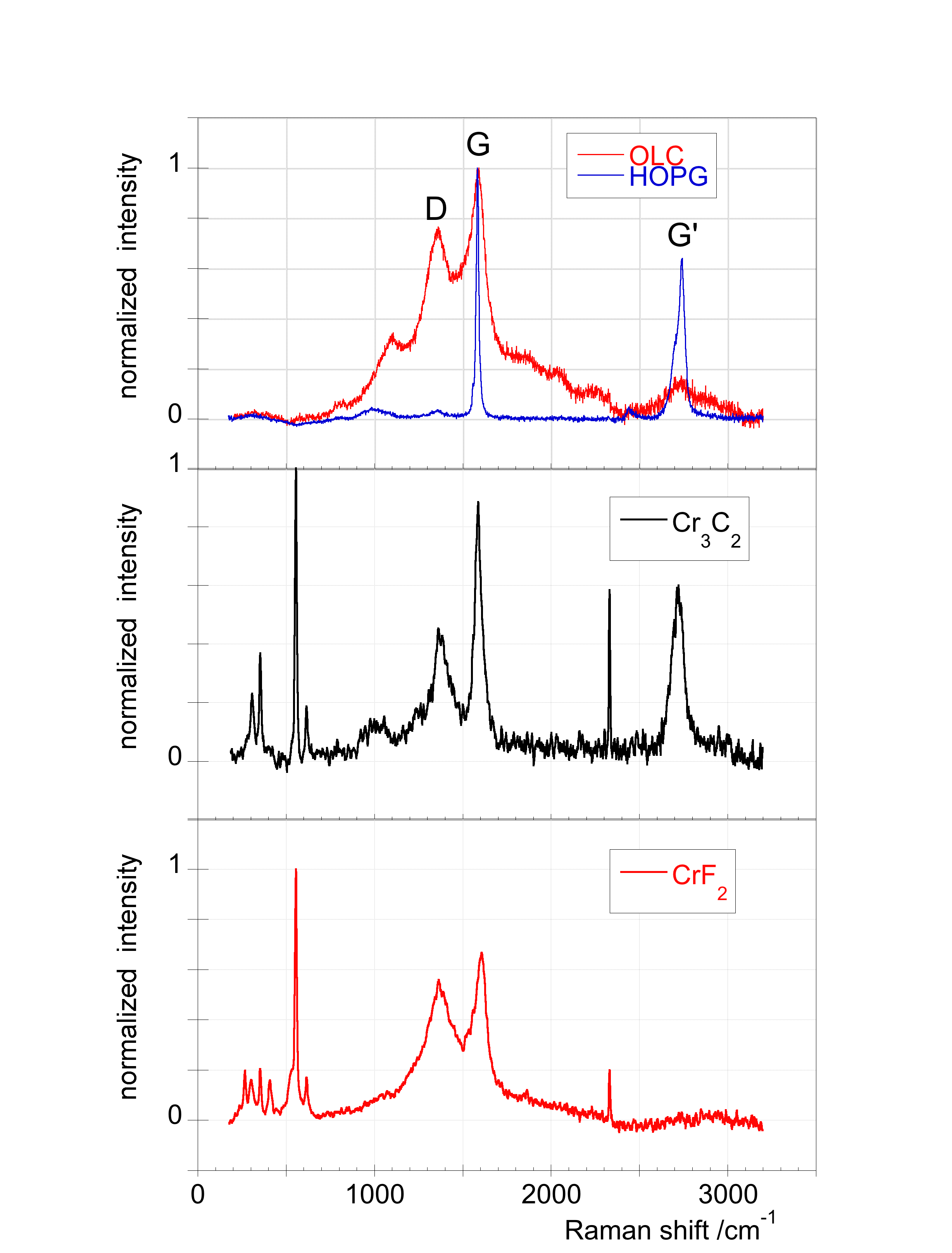
The samples were characterized by making use of a Renishaw inVia Raman Microscope (UK) equipped with Ar laser and blue line was used (λ = 488 nm, 2.54 eV).

Figure S3. microRaman spectra a λ = 488 nm of all ‘as received’ compounds of the electrodes of cell A. From the top: OLC & HOPG, Cr_3_C_2_ and CrF_2_.

- - - 1. HR-TEM


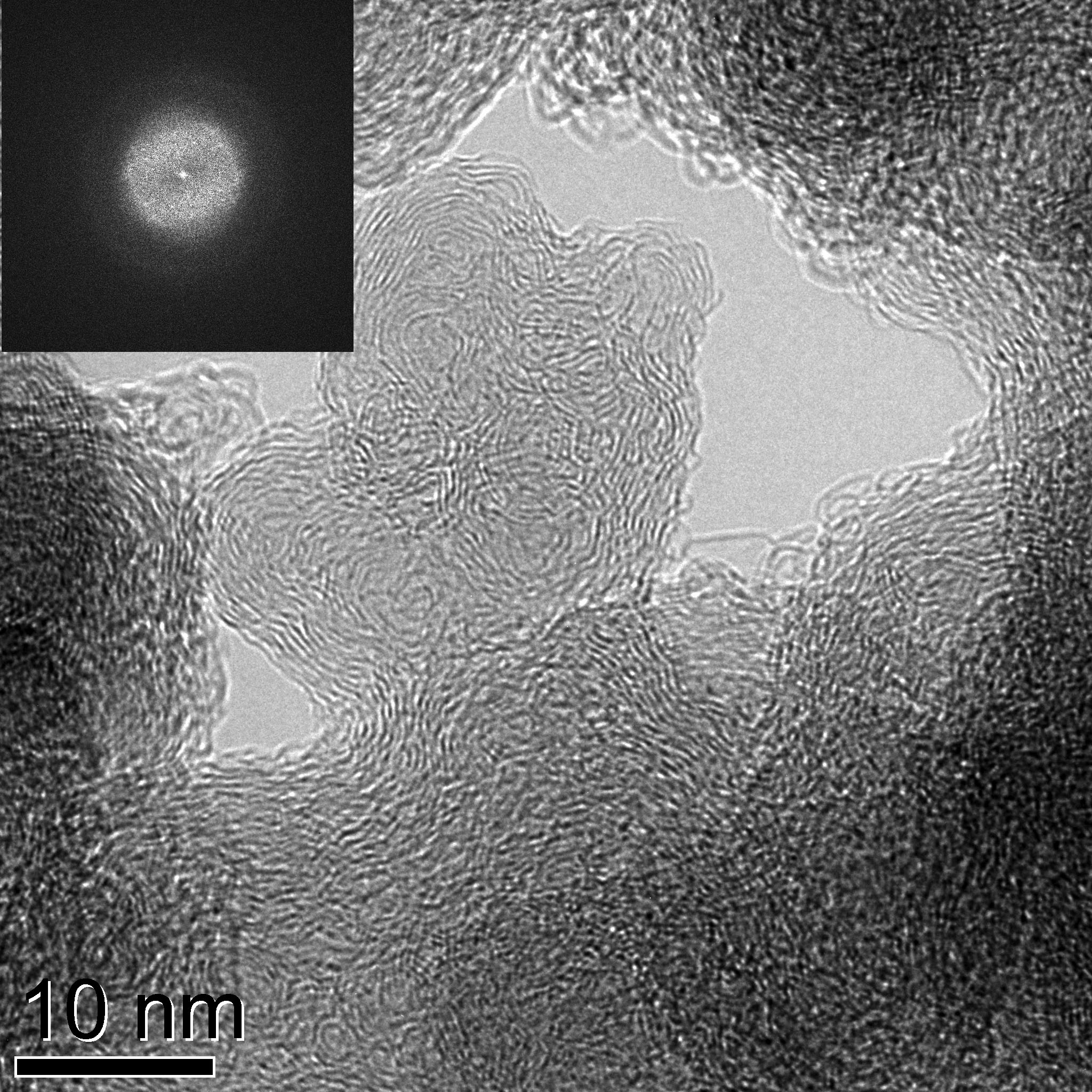
TEM analyses were performed by a JEOL JEM 2200FS field emission transmission electron microscope operating at 200 kV (resolution: 0.19 nm) and Ω type energy filter. Samples for imaging were prepared by ultrasonic dispersion of small amounts of powders in isopropanol and then putting a drop of the dispersion on holey carbon-coated copper grids.

Figure S4. HR-TEM of the ‘as received’ OLC sample. The insetin the top left corner shows the FFT.

The inset shows the FFT. The rings expected for spacing parameters of graphite (c = 0.34 nm and a = 0.20 nm) are very weak indicating a low crystalline sample.

- - - 1. XPS

X-ray photoelectron spectroscopy was carried out by means of Thermo Scientific Theta Probe. The OLC sample, as well as the reference samples, was examined by depositing an isopropanol suspension directly onto a gold foil. The sample was then left to dry for several hours in the load lock chamber. It was analysed before and after Ar^+^ etching in order to obtain a C1s signal free of contamination carbon. C1*s,* O1*s,* valence band and survey spectra of the carbon were measured in the standard emission geometry using a monochromatic Al Kα radiation photon energy 1486.6 eV. The high-resolution spectra were acquired setting two different pass energies (50 eV and 100 eV), while the survey spectra were recorded using pass energy of 200 eV. The energy resolution was determined from the Gaussian width of the C1*s* line of PET and it was found in these experimental conditions equal to 0.8 eV at pass energy of 50 eV. All measurements were carried out at the total pressure of 10^-7^ Pa. The spectra were processed using CASAXPS software (v2.3.15): curve fitting was performed after Shirley background subtraction. The spectra of pure graphite were also collected using the same analysis conditions and the curve fitting parameters, i.e., the full width at half maximum of the peak height (fwhm), the G/L ratio and tail function (T) were constrained to the values acquired on HOPG and diamond. The C1*s* photoemission spectra of *sp^2^* bound carbon were fitted using a Gaussian component that accounts for the instrumental energy resolution together with any chemical disorder, combined with a Lorentzian width that accounts for the finite core hole lifetime associated with the photoionization process. Quantitative analysis was calculated using Scofield’s photoionization cross-sections corrected for the asymmetry factor, intensity energy response function and inelastic mean free-path calculated using NIST approach [[[1]](#endnote-2)].

Only carbon and oxygen were detected on the surface of OLC samples. The comparisons of C1s peak are reported in Figure S5 and in the table S1.


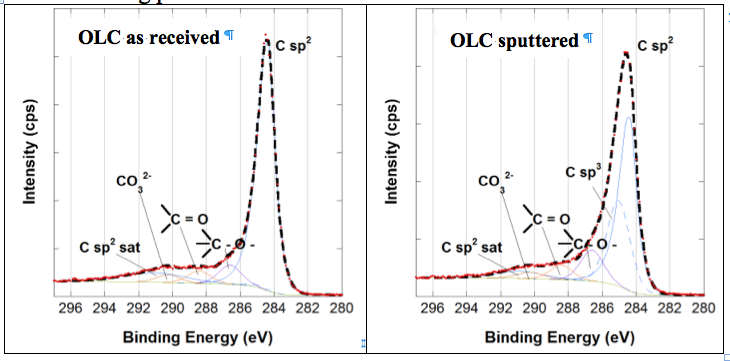


Figure S5. Comparison of XPS C1s spectra of OLC samples ‘as received’ (left side) and after sputtering (right side) .

Table S1. Binding energy values (eV), full width at half-maximum of the peak height. line-shape of the C1s signals and relative weight of the signals in the OLC sample before and after Ar^+^ sputtering.

|  | **C1s (1)**  sp^2^ | **C1s(2)**  sp^3^ | **C1s(3)**  C-O | **C1s(4)**  C=O | **C1s (5)**  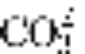 |
| --- | --- | --- | --- | --- | --- |
| **OLC** | 284.4 eV  (0.82)  G/L(85)T(1.5)  84% | 285.0  (1.65)  G/L (30)  1% | 286.6 eV (1.65)  G/L (30)  7% | 288.4 eV (1.65)  G/L (30)  5% | 290.2 eV (1.65)  G/L (30)  3% |
| **OLC sputtered** | 284.4 eV  (0.82)  GL(85)T(1.5)  55% | 285.0  (1.65)  GL (30)  28% | 286.6 eV (1.65)  GL (30)  10% | 288.4 eV (1.65)  GL (30)  5 % | 290.2 eV (1.65)  GL (30)  2% |

As a consequence of Ar^+^ sputtering a change in the shape of C1s is observed with an increase in the component at 285. 0 eV, that can be assigned to sp^3^ carbon. The intensity of the component at 285.0 eV increases with sputtering time.

- - 1. Preparation of electrodes and cell assembly
       1. Electrodes

The electrodes were prepared in a glove box filled with inert and dry atmosphere according to the criterion of establishing the polyphasic coexistence through a close contact among the powder particles. Each powder mixture was prepared by mixing HOPG and OLC with CrF_2_ and Cr_3_C_2_ in weight ratios 1:1:1 and 1:10:10 respectively. Each mixture was ball milled at 50 rpm overnight in a sintered alumina jar with sintered alumina spheres in order to ensure a truly intimate mixing of the phases. Possible modifications to the reactants were checked by XRD. Following the well-established standard procedure, the fine powder mixture was dispersed in acetone and, after evaporation of the solvent, pressed in a stainless steel mould at 0.6 GPa for obtaining cylinders 6 mm diameter x 3 to 5 mm height. The surfaces of electrodes were gently polished in such a way as to be flat and in perfect contact with the electrolyte surfaces.

- - - 1. Cell assembly

The cell holder containing the electrodes, electrolyte and Pt lead wires is machined from a workable alumina rod (Aremco, USA). Two small holes (1 mm dia.) serve as an outlet for the Mo wires and cell out gassing. All the components of the cell were assembled as a sandwich in which the electrolyte is in the middle. The electrolyte is a polished disk of CaF_2_ monocrystal (MolTech GmbH, Germany) (111) oriented, 2 mm thick and 8 mm diameter. The total length of the cells was never greater than 12 mm. Molybdenum wires up to the feedthroughs of the furnace flange realized the electrical leads.

- - 1. Experimental apparatus for the *emf* measurements

The *emf* measurements were carried out in a high-vacuum vertical furnace made of a W mesh resistor from Oxy-Gon Industries Inc., USA. An inconel spring-loaded latticework was utilized for positioning the holder of the electrochemical cell in the isothermal zone of the furnace. The vertical force applied to the cell is maintained at a preset value by a feedback motion device in such a way as to compensate size changes due to the temperature variations. In this way the contact pressure at the electrode/electrolyte interface is independent from temperature. Throughout the experiment the average hydrostatic pressure applied to cell was 12±3 kPa. The temperature was measured by two S-type (Pt/Pt-Rh 10%) thermocouples, which were calibrated against pure Au melting point. Before starting the *emf* measurements, the system was carefully flushed with Ar (O_2_ < 1 ppm, H_2_O < 1 ppm) and then outgassed by following a standard procedure, which does not allow any temperature increase of the cell if the pressure inside the furnace is greater than 1x10^-6^ mbar. The total pressure during the experiment is maintained below 1x10^-7^ mbar. A high impedance differential preamplifier (10^15^ ohm, typical bias current 40 fA) connected to one of the analog input channels of a data logger was used for the *emf* measurements.

- - 1. Procedure adopted for the *emf* measurements and their data acquisition

The total accuracy, *ε*, in reading the *emf* was less than 50 µV. A data logger connected to a personal computer operating on a LabView© based program read the furnace and cell temperatures, pressure, *emf* as well as other signals. The stability of the temperature of the furnace at the set temperature was always within 1 K. The temperature changes throughout the experiments were performed by programmed ramps with a slope of ±3 K min^-1^.

Each thermal cycle on the same cell was in general constituted of about 50 isotherms and each isotherm of 3 hours length was constituted by 150 point acquired. Data in Fig. 1 in the text refer to 4 cycles (about 700 hours) on one out of five cells tested. The thermal cycle is programmed to scan the temperature interval up and down. A LabView© based software performs the off-line elaboration of data files by separating each isotherm and looking for the requirement of the *emf* stationary. This requirement for each isotherm is based on the convergence of the quantity $\chi_{i}/\bar{E}_{i}$ calculated on every *i*-10 points. $\bar{E}_{i}$ is the average of the *emf* values and $\chi_{i}$ its standard deviation. If the ratio converges to a value, which is within 10*ε*, the last $\bar{E}_{i}$ is attributed to that isotherm with error bar equal to ±$\chi_{i}$. The isotherm is discarded if this condition is not fulfilled. The temperature attributed to the accepted *emf* value is $\bar{T}\pm\chi_{T}$ where $\bar{T}$ and $\chi_{T}$ are respectively the average temperature and the related standard deviation on the whole isotherm.

- - 1. Analysis of electrodes before and after experiment
       1. XRD

The intensity full-scale range of the XRD pattern of the electrode with HOPG “before” (bottom-right panel) was reduced of one order of magnitude to make visible the features of Cr_3_C_2_ and CrF_2_ with respect to the high intensity of the main peak of HOPG. Due to improved crystallization of CrF_2_ with respect HOPG, the XRD pattern of the same electrode “after” can be shown (up-right panel) with its own full-scale range.


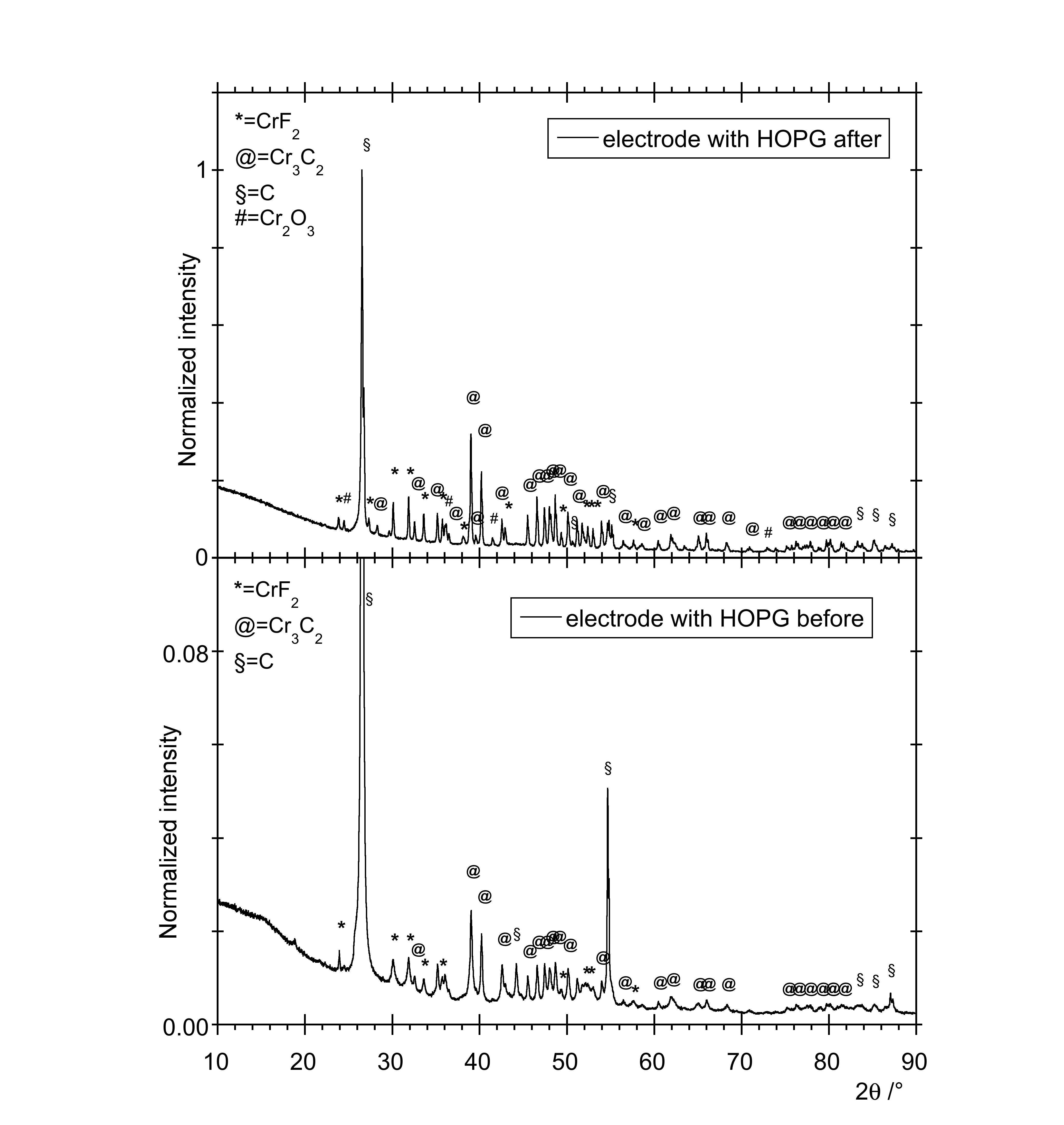

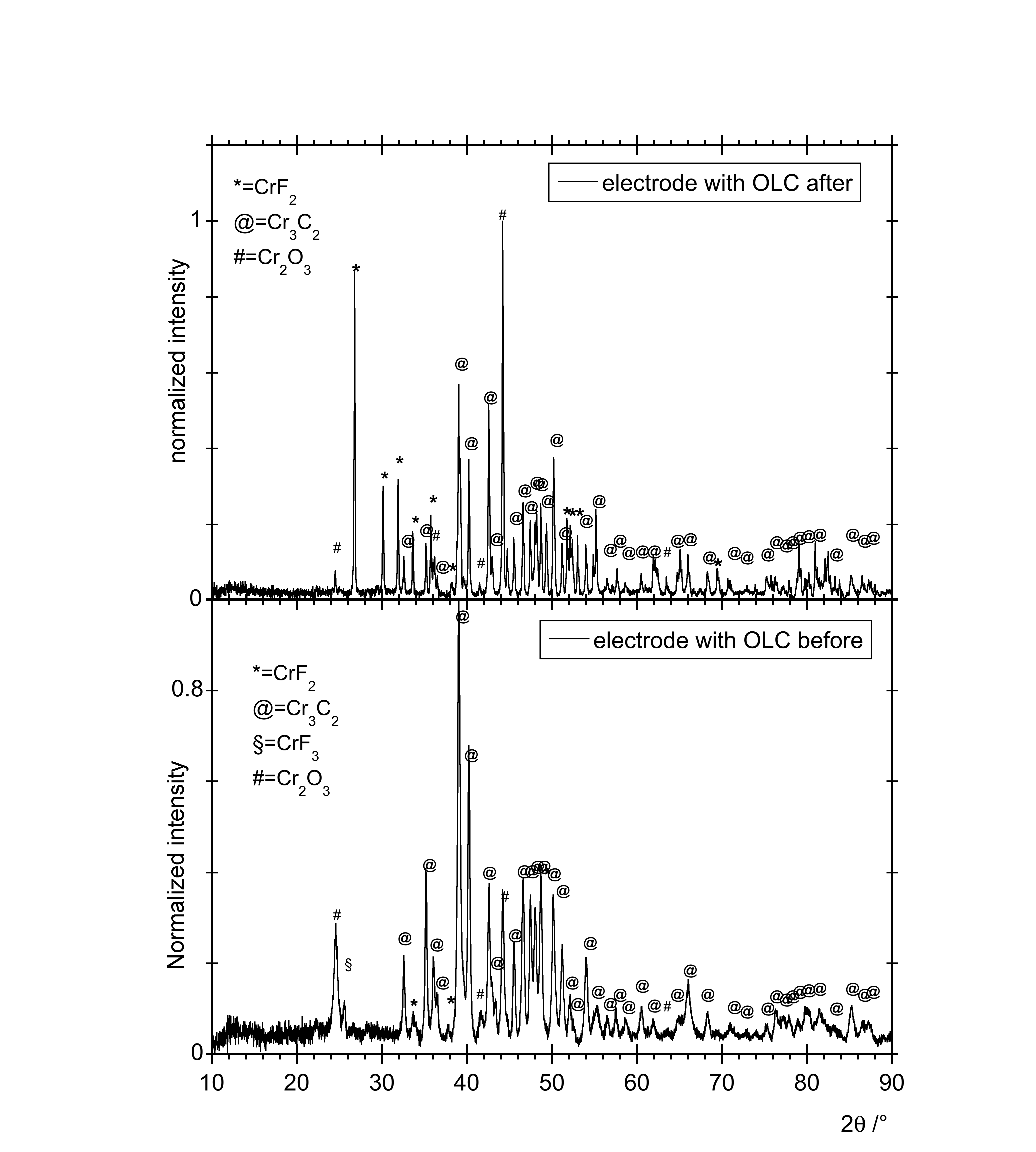


Figure S6. XRD comparison of the electrode powder mixtures containing OLC before and after an experiment. Due to the amorphous nature of OLC and their low content in the mixture, no features of them were detected. No evidence of new and/or disappeared phases was found.

Figure S7. XRD comparison of the electrode powder mixtures containing HOPG before and after an experiment. The features of HOPG carbon were clearly detected in both cases. No evidence of new and/or disappeared phases was found.

- - 1. microRaman spectroscopy (mR)

mR spectroscopy confirms further that no other chemical species were formed during the experiment because the spectra of both the electrodes did not change with exception of some differences in the intensity that are meaningless.


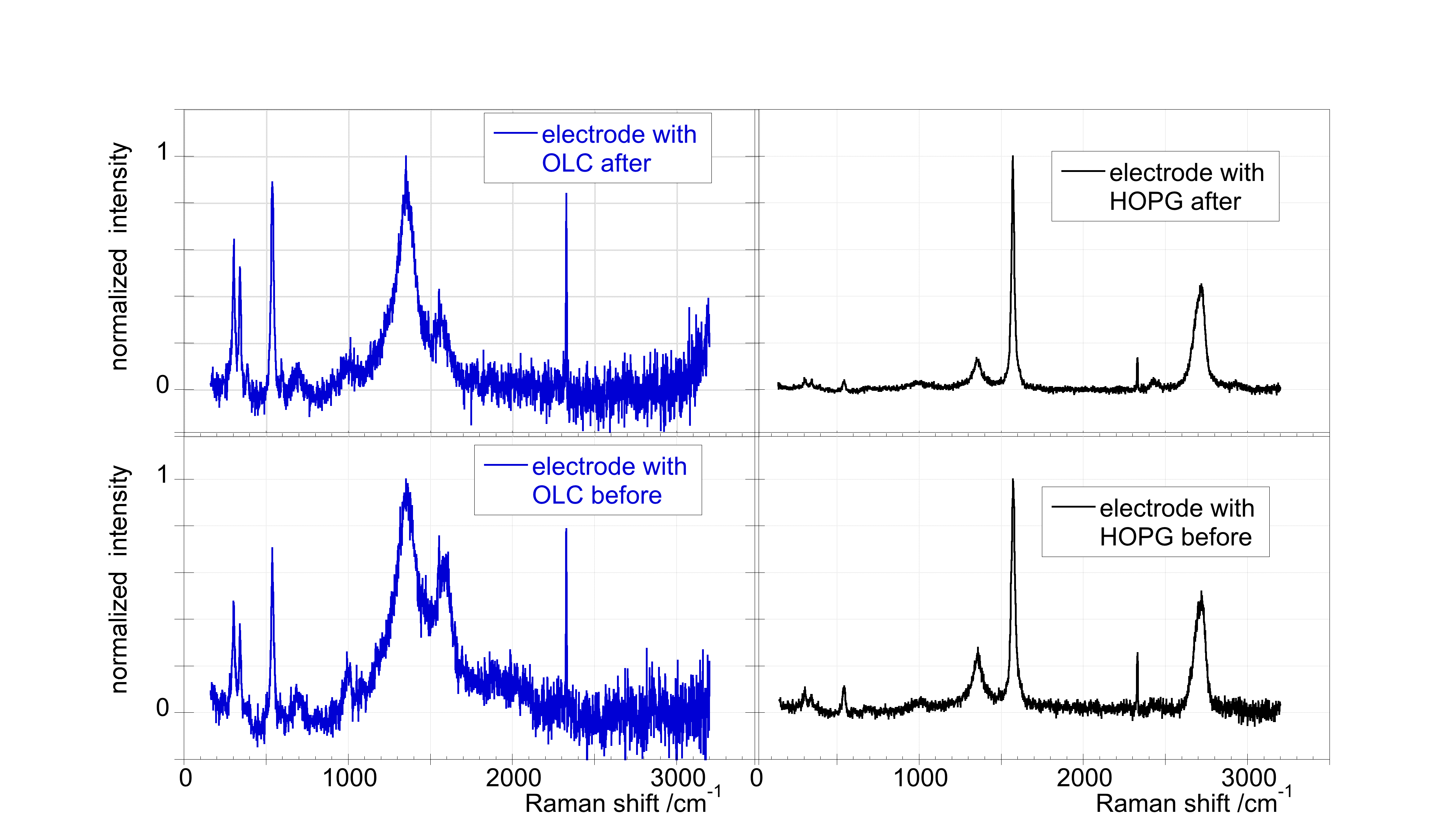


Figure S8. Comparison of the microRaman spectra of the electrode powder mixtures containing OLC (left side) and HOPG (right side) before and after an experiment. No evidence of changes was found.

- - 1. EELS

This technique, as well as the XPS below, was utilized to determine the sp^2^/sp^3^ ratio in OLC by utilizing diamond and HOPG powder as standards for full sp^3^ and sp^2^, respectively. The carbon-K ionization edge (onset at 284 eV) were collected on a JEOL JEM-2200FS only on OLC hanging out in the grid holes, to avoid interferences from the carbon support film of the grids or from other parts of the samples. The acquisition of spectra was in “magic collection angle β” mode [[[2]](#endnote-3)] with β ≅ 1.5 mrad for carbon at 200 kV. The convergence angle α < β ensures that the effect due to the sample orientation gives a negligible error, which was found lower than the statistical one as verified on reference graphite. The samples were grinded and dispersed in isopropanol, and then deposited on holey carbon grids. The measurements have been carried out in many different regions of the samples to improve the statistics. Panel A of the figure below shows the EELS spectrum of the “as received” OLC sample (blue line) compared with OLC in the electrode after the experiment, the reference HOPG (black line) and amorphous carbon (green line). Panel B shows how the sp^2^/sp^3^ ratio was determined. This was done by the curve deconvolution [[[3]](#endnote-4)] and Gaussian fit of the main σ* peak in the region 291-293 eV (green line). The π* contribution (red line) was obtained by subtraction of σ* peak from the total curve in the interval 280-292 eV. The sp^2^/sp^3^ ratio was evaluated by the ratios between the π* peak area of OLC samples as well as amorphous carbon and π* peak area of reference graphite. The sp^2^ carbon coordination
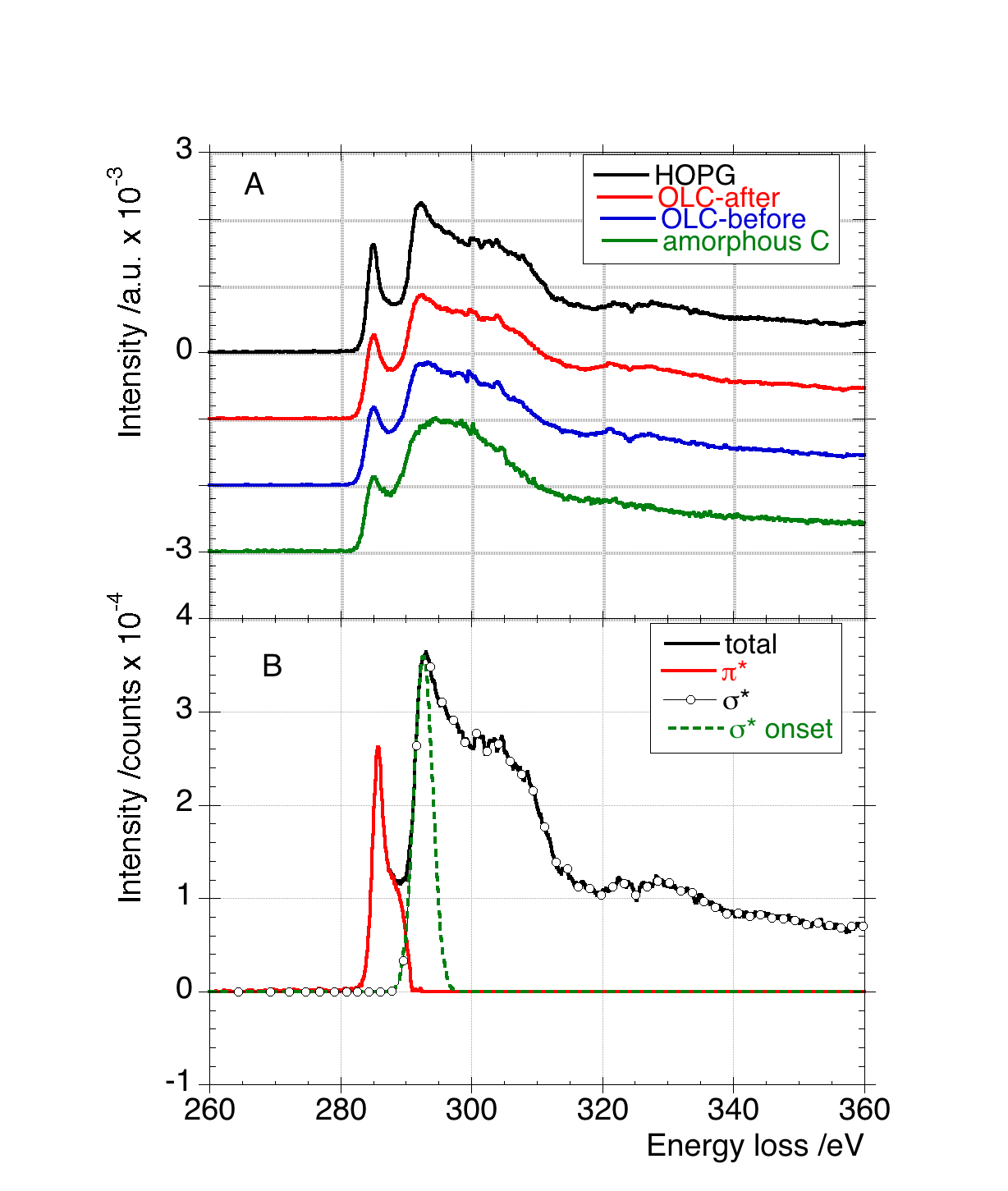
values were found 82±3 %, 86±3 % and 69±8 % for OLC before and after and amorphous carbon, respectively. The result points to a graphitization of OLC towards multi-shell ordered structures in the process, as confirmed by clearer reflections in the FFT of the HRTEM images after the experiment, corresponding to the expected graphite reflections. The planes are bended to form the spherical structures seen in the HRTEM images of Fig. 8B in the text.

Figure S9.

A. EELS spectra of OLC before and after an experiment compared with two references for sp^2^ content HOPG and amorphous carbon.

B. Evaluation of the sp^2^/sp^3^ ratio in the OLC samples.

- 1. **Derivation of eq 18**

The relationship between $\sigma$ and $\gamma$ reads,

$\gamma=\sigma+A\frac{d_{s}\sigma}{d_{s}A}$ (1)

where *A* denotes the area of the surface and the subscript *s* reminds us that the variation is performed by stretching. Let us consider a one–component nano particle of radius *r* in equilibrium with its vapour at constant temperature and volume. At equilibrium *dF* = 0, with *F* being the Helmholtz free energy:

$dF=\left(\mu_{B}-\mu_{V}\right)dN+\sigma\frac{\partial A}{\partial N}dN+\frac{\partial F_{r}}{\partial N}dN=0$ (2)

that is

$\mu_{V}=\mu_{B}+\sigma\frac{\partial A}{\partial N}+\frac{\partial F_{r}}{\partial N}$ (3)

In this equation $\mu_{B}$ and $\mu_{V}$ are the chemical potentials of the components in the Gibbs model system ($r\to\infty$) and in the vapour, respectively; $F_{r}<0$ is the relaxation energy that depends on deformation of both surface and bulk of the particle. Accordingly, the surface energy (*σ*) refers to unrelaxed surface, i.e., to a particle of infinite radius. Moreover, the Laplace equation gives the pressure difference between the two phases (particle-vapour) as,

$P_{i}-P_{ex}=\frac{2\gamma}{r}$ (4)

where *P_i_* is the pressure in the particle and *P_ex_* the external pressure. It is evident from eq 4 that the particle is in a state of compression for $\gamma>0$. Consequently, the volume change of the particle (on going from *P_ex_* to *P_i_*) owing to the surface tension can be computed by means of the compressibility, *κ*, as $\frac{\Delta V}{V}=-\kappa\left(P_{i}-P_{ex}\right)=-\kappa\frac{2\gamma}{r}$, with *V* being the particle volume.

Let us now compute the relaxation energy term, $F_{r}$. To this purpose, it is considered a closed system made up of particle and vapour phases at constant total volume. One estimates the energy change for the relaxation of the surface through stretching which entails the pressure difference given by eq 4. The whole energy change is given by the sum of the following contributions: $F_{r}=\Delta F_{V}+\Delta F_{1}+\Delta F_{2}$ where $\Delta F_{V}$ is the term linked to the vapour, $\Delta F_{1}$ is the energy term due to the “volume” strain of the particle and $\Delta F_{2}$ is the surface tension contribution. For uniform compression the $\Delta F_{1}>0$ term is given by $\Delta F_{1}=\frac{1}{2}\kappa V\left(P_{i}^{2}-P_{ex}^{2}\right)=\frac{\gamma\kappa V}{r}\left(\frac{2\gamma}{r}+2P_{ex}\right)$, where eq 4 was employed. The change of the energy of the vapour is given by $-P_{ex}\Delta V_{V}=P_{ex}\Delta V=-P_{ex}V\kappa\frac{2\gamma}{r}$. Furthermore, the energy gained by the system due to the work done by the surface tension reads: $\Delta F_{2}=\gamma A\frac{\Delta A}{A}=\frac{2}{3}\gamma A\frac{\Delta V}{V}=-\frac{4}{3}A\kappa\frac{\gamma^{2}}{r}$. The relaxation energy is computed eventually as ($A=4\pi r^{2}$):

$F_{r}=\frac{\gamma\kappa V}{r}\left(\frac{2\gamma}{r}+2P_{ex}\right)-P_{ex}V\kappa\frac{2\gamma}{r}-\frac{16}{3}\pi r\kappa\gamma^{2}=\frac{8}{3}\pi r\kappa\gamma^{2}-\frac{16}{3}\pi r\kappa\gamma^{2}=-\frac{8}{3}\pi r\kappa\gamma^{2}$ (5)

It is instructive to verify that eq 5 is in fact the energy change linked to the generation of the excess hydrostatic pressure of eq 4. The relation holds:

$F_{r}=\int_{\Delta P=0}^{\Delta P=2\gamma/r} \Delta PdV=-\kappa V\int_{0}^{2\gamma/r} \Delta Pd\Delta P=-\frac{8}{3}\pi r\kappa\gamma^{2}$ , (6)

which coincides with eq 5. By inserting the $F_{r}$ expression in eq 3 and considering that for the spherical shape $\frac{\partial A}{\partial N}=\frac{\partial A}{\partial V}\frac{\partial V}{\partial N}=\frac{2V}{r}$ and $\frac{\partial F_{r}}{\partial N}=\frac{V}{4\pi r^{2}}\frac{\partial F_{r}}{\partial r}$ , *V* being the molar volume of the solid, one gets

$\mu_{V}=\mu_{B}+\sigma\frac{2V}{r}-\frac{2V}{3r^{2}}\kappa\gamma^{2}$ (7)

Therefore, the chemical potential difference between the components in the particle and in the bulk phase is equal to [[[4]](#endnote-5)]

$\Delta\mu_{s}=\frac{2V}{r}\left(\sigma-\frac{\kappa\gamma^{2}}{3r}\right)$ (8)

where the subscript *s* emphasises that this contribution is entirely due to the interface.

- 1. **Derivation of eq 22**

The integration of equation $\left(\frac{\partial U}{\partial V}\right)_{T}=T\left(\frac{\alpha}{\kappa}\right)-P$ gives at constant temperature:

. (9)

Due to the definition of the isothermal compressibility
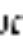
, the volume *V* can be written as $V=V_{0}e^{-\kappa P}$ and the term $-\int_{V_{0}}^{V} PdV$ becomes:

$-\int_{V_{0}}^{V} PdV=V_{0}\kappa\int_{0}^{P} Pe^{-\kappa P}dP=\frac{V_{0}}{\kappa}\left[1-\left(1+\kappa P\right)e^{-\kappa P}\right]$ (10)

being $\int xe^{ax}dx=\frac{e^{ax}}{a^{2}}\left(ax-1\right)$. By substituting eq 10 in eq 9, eq 22

$$\Delta U=\left(U-U_{0}\right)_{T}=\frac{V_{0}}{\kappa}\left\{\left[1-\left(1+\kappa P\right)e^{-\kappa P}\right]+\alpha T\left(e^{-\kappa P}-1\right)\right\}$$

is obtained.

1. . Gries, WH (1996) A universal predictive equation for the inelastic mean free path lengths of x-ray photoelectrons and auger electrons. Surf. Interface Anal. 24: 38–50. [↑](#endnote-ref-2)
2. . Egerton RF (1996) Electron energy-loss spectroscopy in the electron microscope. 2nd Ed., Plenum Press, New York. [↑](#endnote-ref-3)
3. . Verbeeck J, Bertoni G (2009) Deconvolution of core electron energy loss spectra Ultramicroscopy 109: 1343-1352. [↑](#endnote-ref-4)
4. . Shuttleworth R (1950) The Surface Tension of Solids. Proc Phys Soc (London) A 63: 444-457. [↑](#endnote-ref-5)
